# Optimization of Random Phase Approximation Calculations for Improved Energies of Molecules, Solids, and Surfaces

**DOI:** 10.1021/acs.jctc.6c00537

**Published:** 2026-07-30

**Authors:** Neung-Kyung Yu, Johannes Voss, Andrew J. Medford

**Affiliations:** † School of Chemical & Biomolecular Engineering, 1372 Georgia Institute of Technology, Atlanta, Georgia 30332, United States; ‡ SUNCAT Center for Interface Science and Catalysis, 497525 SLAC National Accelerator Laboratory, Menlo Park, California 94025, United States

## Abstract

We present an optimized random phase approximation method (optRPA26) that significantly improves upon conventional RPA through an optimized choice of reference orbitals and energy components, rather than a modification of the RPA correlation functional itself. The method employs an empirically constructed hybrid functional to generate DFT orbitals to evaluate the RPA correlation energy, which is then scaled by a constant. Comprehensive benchmarks across molecules, bulk solids, and surface systems demonstrate that optRPA26 consistently achieves high accuracy, with mean absolute errors of 0.05 eV for W4-11-RE reaction energies, 0.07 eV for cohesive energies, 0.09 eV for metal oxide formation energies, 0.11–0.12 eV for adsorption of small molecules on metals, and 0.06 eV for adsorption on oxides. In addition, optRPA26 correctly captures phase stability in metal oxides and magnetic metals. The optRPA26 approach can be run using standard RPA implementations, highlighting its potential as a general-purpose reference method that can accurately capture covalent, ionic, metallic, and van der Waals bonding in molecules, solids, and interfaces.

## Graphical abstract

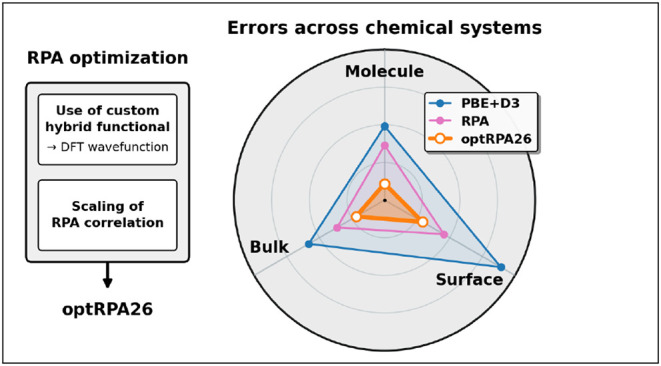

## 1 Introduction

Density functional theory (DFT) is the most widely used approach for quantum mechanical (QM) simulations in physics, materials science, and chemistry.^1^ Many problems in applied sciences require computing properties of systems with ∼100 atoms or more, often with periodic or mixed boundary conditions. DFT is capable of performing these simulations within a practical time frame due to its relatively low computational cost and the wide availability of efficient implementations. However, the lack of a universally accurate exchange-correlation (xc) functional leads to errors that can greatly exceed the “chemical accuracy” (1 kcal mol^–1^ or 0.043 eV) needed for quantitative predictions of reaction rates.^2^ For transition metal systems, the uncertainty is even greater from both experimental and theoretical points of view, so the chemical precision of the transition metals (3 kcal mol^–1^ or 0.130 eV) has been defined more loosely.^3–5^ Although numerous specialized functionals exist, the lack of systematic improvement in functionals makes it difficult to determine a priori which approximation will be most accurate for a particular chemical system. The development of an xc functional that achieves chemical accuracy across all types of systems—including atoms, molecules, liquids, ionic solids, metals, and interfaces—is a grand challenge in the field of computational chemistry.^6,7^ The problem is particularly acute in surface science in catalysis, where there is currently no method capable of achieving chemical accuracy for adsorption at metal surfaces.^8^

Wave function methods offer a more accurate alternative to DFT. However, their high computational cost and limited availability of periodic implementations make them impractical for many problems in materials science and solid-state physics. Nevertheless, two wave function approaches have become increasingly efficient and have been applied to solid-state problems with periodic boundary conditions. The first is coupled cluster with singles, doubles, and partial triples (CCSD­(T)), widely regarded as the gold standard for chemical calculations, achieving chemical accuracy in most systems where it can be applied.^9,10^ Although CCSD­(T) is computationally very expensive, with formal $O\left(N^{7}\right)$ scaling^9,10^ more efficient approaches have been developed.^11^ Specifically, recent progress in local approximations to CCSD­(T) led to the development of variants such as PNO-CCSD­(T),^12^ DLPNO-CCSD­(T),^13^ and LNO-CCSD­(T).^14–16^ In practice, CCSD­(T) is primarily applied for gas molecules since most implementations do not support periodic boundary conditions. However, periodic CCSD­(T) implementations have recently become available.^17^ Several groups have successfully applied local CCSD­(T) methods (both periodic and nonperiodic) to calculate highly accurate adsorption energies on oxide surfaces.^8,18–21^ Despite this progress, the implementation of partial triples (T) remains problematic for metallic systems due to energy divergence, also known as the infrared catastrophe.^17,22^ Only very recently, modified approximations to the triples (such as ring-CCSDT and cT) have been proposed to address this limitation.^23–26^

In addition, quantum Monte Carlo (QMC) methods, such as diffusion Monte Carlo (DMC), have been applied even more widely to problems in solid-state physics and surface science.^27–29^ In principle, DMC is an exact method, but its accuracy is typically limited by practical factors such as the number of moves for statistical sampling, time step size, specialized pseudopotentials, and slow convergence of Brillouin zone sampling via supercell extrapolation, as well as the fixed-node approximation.^27,28,30,31^ Although generally much more accurate than DFT, statistical error bars on practical quantities such as formation energies and adsorption energies often exceed chemical precision.^29,32,33^ Due to the lack of highly accurate and practical computational methods for surfaces, there are only a few well-established reference datasets, such as the CE39 ^34^ or ADS41 dataset^35^ of experimental and DFT adsorption energies on simple metal surfaces.

Another strategy to improve upon DFT methods is the use of embedding schemes that combine low-level and high-level QM methods. The high-level method is typically applied to small clusters, whereas the low-level method is used to describe larger systems, such as periodic models. Embedding approaches have been applied to describe adsorption on oxide surfaces using MP2 or CCSD­(T) as the high-level method^18,19,21,36–38^ and on metal surfaces using hybrid functionals, random phase approximiation (RPA), or wave function theories as the high-level method.^39–43^ In particular, the *PBE+D3/M06* approach is an embedding scheme that combines cluster (at hybrid M06 level) and periodic models (at PBE+D3 level). This approach has been reported as one of the most accurate methods for adsorption energies on metals and is therefore used as a comparison in this benchmarking study. Despite the potential for high accuracy in embedding methods, they generally require specialized software and expertise, and are not widely used in the computational materials science community.

Returning to standard DFT, the concept of Jacob’s ladder of density functional approximations describes the physical hierarchy of density-based quantum chemical methods into five levels.^44^ Different levels of DFT methods are positioned on each rung, starting with the local density approximation (LDA) on the first rung, followed by the generalized gradient approximation (GGA), meta-GGA, and hybrid functionals. Unlike those on the lower rungs, the methods on the fifth rung consider unoccupied orbitals for *ab initio* calculation of correlation energy, with the RPA located at this level. RPA correlation energies are computed with help of the adiabatic connection fluctuation–dissipation theorem (ACFDT), where the interacting density response function is approximated at the RPA level. The ACFDT was established over 40 years ago,^45^ but ACFDT-RPA correlation energies have not become a standard choice. While RPA is generally applicable to solids, it remains computationally expensive and exhibits limited accuracy, particularly for molecular systems, despite its physical soundness.^46,47^

There have been continued efforts to improve the accuracy of RPA. One strategy involves correcting the relatively less accurate short-range RPA correlation using the short-range LDA or GGA correlation. This concept underlies the RPA+^48^ and the generalized RPA+,^49^ which have been applied to atomic or molecular systems. An alternative approach introduces a second-order screened exchange (SOSEX) term to the RPA energy, leading to the RPA+SOSEX method.^50^ Similarly, singles excitation (SE) and renormalized singles excitation (rSE) contributions have been proposed, resulting in RPA+SE, RPA+rSE, and RPA+SOSEX+rSE.^51,52^ Although these corrections improve the accuracy of RPA, their overall performance remains limited and often unbalanced.^51,53^ Other beyond-RPA methods, based on time-dependent DFT, include the renormalized adiabatic local density approximation (rALDA) and the renormalized adiabatic Perdew–Burke–Ernzerhof (rAPBE) xc kernels.^54^ These approaches show even higher accuracy across different classes of systems.^55^ Despite the promise of these approaches, most are not widely available in standard electronic structure packages, and thus require specialized expertise or software, limiting their application in practice.

In contrast, the approach introduced in this work focuses on optimizing the standard RPA calculation, often referred to as direct RPA,^56^ to achieve substantially higher accuracy at the cost of generating hybrid-level wave functions, while leveraging widely implemented RPA algorithms. In standard RPA, the total RPA energy is typically evaluated on PBE orbitals (referred to as RPA@PBE), but RPA calculations using a hybrid PBE0 functional (RPA@PBE0) have also been reported.^40,46^ In a prior study, we used a global PBE hybrid with 50% exact exchange (EXX) to incorporate nonlocal interactions and then scaled the RPA correlation energy,^57^ which we refer to here as optRPA24. This approach yielded accurate results for molecular atomization and reaction energies, but suffered from energy convergence issues with respect to k-point sampling in the adsorption energies on metallic surfaces. In addition, the hybrid functional used for the wave functions did not yield the correct magnetic properties for bulk magnetic metals. Here, we use a long-range corrected (LC) custom hybrid functional that includes 25% EXX only in the long-range to construct DFT orbitals. The RPA correlation energy is then scaled by a constant, which is fitted using the W4-11 dataset of highly accurate molecular atomization energies. Finally, short-range PBE correlation is applied when evaluating the non-RPA correlation part of total energy, which reduces the optimal scaling constant to ∼1 and improves energy convergence. The approach of scaling the RPA correlation energy has been reported in LC double-hybrid frameworks, where the total energy includes long-range EXX and long-range RPA correlation.^58,59^ Our optimized RPA approach, which we refer to as optRPA26, can be run using a standard VASP installation (compiled with the Libxc library^60^) without modification to the source code, making it a practical alternative to more specialized approaches. Here, we present the details of the formulation and benchmark it against a diverse dataset of molecules, bulk solids, and surfaces, demonstrating that optRPA26 can serve as a highly accurate reference method for general systems in computational chemistry and catalysis.

## 2 Methods

Details of the benchmark datasets and electronic structure calculations are summarized here. Additional details, including spreadsheets of benchmark results, input files, and example calculations, are provided in the Supporting Information (SI).

### 2.1 Benchmark Datasets

The following benchmark datasets were used as ground-truth reference values in this work. Additional details of data curation, reference values, and computational protocols are provided in the Details of Benchmark Datasets section of the SI.

- Molecular atomization energies (W4-11):^61^
  - 140 atomization energies of small molecules and radicals, calculated using highly accurate W4 theory.^62^
  - We considered 124 molecules with less multireference character (TAE_nonMR124 subset^61^), which are expected to be more representative of molecules relevant to catalysis and provide a more balanced dataset for optimization, because systems with strong multireference character are more difficult to treat reliably even with high-level methods such as CCSD­(T) and RPA-based approaches. A list of excluded molecules can be found in ref 63.
- Molecular reaction energies (W4-11-RE):^64^
  - 11,247 reaction energies derived from the W4-11 dataset.
  - We selected only reactions involving molecules in TAE_nonMR124, resulting in 8,868 reactions.
- Molecular barrier heights (BH76):^65–67^

  - 76 forward and reverse barrier heights for hydrogen transfer, heavy-atom transfer, nucleophilic substitution, unimolecular, and association reactions.
- Molecular reaction energies (BH76RC):^67^
  - 30 reaction energies derived from the BH76 dataset.
- Reactions of organometallic transition metal (TM) complexes (MOBH29):^68–71^

  - A revised version of MOBH35.^68^
  - 29 forward and 29 backward barrier heights, as well as 29 reaction energies.
- Noncovalent interaction energies (S19):
  - We selected 19 interaction energies from the S66 dataset.^72^
- Bulk solid datasets:
  - 24 lattice constants, 24 bulk moduli, and 24 atomization energies of nonoxides.^73–75^

  - 23 oxide formation energies.^76,77^

  - Magnetic moments of magnetic solids (Fe, Co, and Ni).^78^
  - Relative stability of bulk phases of Co (hcp, fcc, and bcc phases), MoO_3_ (α and β phases), and TiO_2_ (rutile and anatase phases).
- Surface datasets:
  - 27 experimental adsorption energies on transition metal surfaces derived from ADS41.^34,35,79^

  - 7 surface reaction energies on oxide surfaces (5 CCSD­(T)-level calculation values^8,20^ and 2 experimental values^21^).
  - Surface energies of four transition metal surfaces with (111) facet.^80^

### 2.2 Computational Details

All periodic calculations were performed using the Vienna *Ab initio* Simulation Package (VASP) version 6.3.2, employing projector-augmented wave (PAW) pseudopotentials.^81–84^ GW-type PAW pseudopotentials (version 54) were used throughout. Specifically, the pseudopotentials recommended by VASP^85^ were employed, except for C, N, O, and F, for which “GW_new” pseudopotentials were used. Another exception was for metal slab calculations, where GW pseudopotentials with the fewest valence electrons were selected. The GW pseudopotentials used for the metal slabs do not include semicore s and p states in Ni, Cu, Rh, Pd, and Pt, while those for Ru and Ir include them.

Spin polarization was considered in all molecular datasets (see Section S2.2 of the SI) and, in solids, only for magnetic materials, including Fe, Ni, and Co–containing systems and alkali-metal superoxides (NaO_2_, KO_2_, RbO_2_, and CsO_2_). The fast Fourier transform (FFT) grids for GGA (PREC) and exact exchange (PRECFOCK) were set to “Accurate” and “Normal”, respectively, with the exception of calculations for nonoxide bulk systems (see Section S2.3 of the SI).

For DFT calculations, the following dispersion-corrected DFT functionals were used: PBE+D3 (GGA),^86,87^ r2SCAN+rVV10 (meta-GGA),^88–90^ and HSE06+D3 (hybrid).^87,91^ All other calculation parameters were identical to those used for RPA calculations, unless otherwise specified. Structural relaxations were performed using PBE+D3, and the same structures were used for single-point calculations with all functionals. Initial structures for the oxide formation dataset were obtained from the Materials Project.^92^

For RPA calculations, a cubic-scaling algorithm was used^93^ with a frequency integration grid containing 24 points. For optRPA26, we used an empirically constructed custom hybrid functional, LC-srPBEx25, to generate DFT orbitals for evaluating the RPA correlation energy. This functional uses a long-range corrected hybrid scheme with a range separation parameter (μ) of 3.0 Å^–1^ and 25% exact exchange. The GGA xc component was described using the short-range PBE (srPBE) functional^94–96^ with the default range separation parameter of 0.9449 Å^–1^ (0.5 Bohr^–1^) for both exchange (μ) and correlation (μ_*c*_) via the Libxc library (version 7.0.0).^60^ The LC-srPBEx25 functional includes 25% long-range (lr) exact exchange, 75% srPBE exchange, and 500% srPBE correlation (see Section 4.1 in the Discussion), as defined below. This empirically constructed functional is used solely to generate Kohn–Sham orbitals, which have been found to serve as suitable inputs for RPA energy evaluation, and is not used to calculate DFT energies. Its exchange-correlation energy is calculated as

1 $$E_{\mathrm{xc},\mathrm{LC‐srPBEx25}}=\left(0.25\cdot E_{x,\mathrm{EXX}}^{\mathrm{lr},\mu =3.0{\;Å}^{-1}}+0.75\cdot E_{x,\mathrm{srPBE}}^{\mathrm{sr},\mu =0.94{\;Å}^{-1}}\right)+5.00\cdot E_{c,\mathrm{srPBE}}^{\mathrm{sr},\mu_{c}=0.94{\;Å}^{-1}}$$

where $E_{x,\mathrm{EXX}}^{\mathrm{lr},\mu =3.0{\;Å}^{-1}}$ is long-range exact exchange, $E_{x,\mathrm{srPBE}}^{\mathrm{sr},\mu =0.94{\;Å}^{-1}}$ is short-range PBE exchange, and $E_{c,\mathrm{srPBE}}^{\mathrm{sr},\mu_{c}=0.94{Å}^{-1}}$ is short-range PBE correlation.

The total energy in the standard RPA calculation is defined as

2 $$E_{\mathrm{total}}^{\mathrm{RPA}}=E_{\mathrm{HXX}}+E_{c,\mathrm{RPA}}$$

where *E*_HXX_ is HF energy (HF@PBE) and *E*_c,RPA_ is the RPA correlation energy (RPA_c_@PBE), both evaluated using PBE orbitals. In optRPA24,^57^ the previous version of optRPA26, the total energy is defined as

3 $$E_{\mathrm{total}}^{\mathrm{optRPA24}}=E_{\mathrm{HXX}}+r_{c}\cdot E_{c,\mathrm{RPA}}$$

where *E*_HXX_ (HF@PBEx50) and *E*_c,RPA_ (RPA_c_@PBEx50) are evaluated using PBEx50 (a PBE global hybrid with 50% EXX) orbitals, and *r*_*c*_ (= 1.180) is a scaling constant for *E*_c,RPA_. In optRPA26, the total energy is defined as

4 $$E_{\mathrm{total}}^{\mathrm{optRPA26}}=E_{\mathrm{optHXX}}+r_{c}\cdot E_{c,\mathrm{RPA}}$$

where *E*_optHXX_ is the modified HXX energy that includes a non-RPA correlation contribution of the total energy. Both *E*_optHXX_ (optHXX@LC-srPBEx25) and *E*_c,RPA_ (RPA_c_@LC-srPBEx25) are evaluated using the same LC-srPBEx25 orbitals. Each term in eq 4 is obtained from a separate single-point calculation. The xc part of optHXX consists of 100% full-range EXX (*E*_x,EXX_) and 20% srPBE correlation $\left(E_{c,\mathrm{srPBE}}^{\mathrm{sr},\mu_{c}=1.89{\;Å}^{-1}}\right)$ with a range separation parameter for DFT correlation of 1.8897 Å^–1^ (= 1.0 Bohr^–1^):

5 $$E_{\mathrm{xc},\mathrm{optHXX}}=E_{x,\mathrm{EXX}}+0.20\cdot E_{c,\mathrm{srPBE}}^{\mu_{c}=1.89{\;Å}^{-1}}$$

The srPBE correlation was introduced to reduce *r*_*c*_ for optRPA26 (from 1.114 to 1.02), which improves energy convergence with respect to k-point sampling (see Section 4.3 in the Discussion). We did not include a correction to the non-RPA correlation part for partial occupancies,^73^ as it did not help energy convergence (Figure S9). Although inclusion of this term is formally more rigorous,^48,73^ we found that it did not improve the numerical convergence relevant to the present calculations. In particular, the correction term is highly sensitive to the smearing width and becomes negligible at the small smearing width (0.01 eV) used here.

For RPA calculations, we applied a plane-wave energy cutoff (*E*_cutoff_) correction term Δcutoff for all calculations, except for molecular datasets and nonoxide bulk solids, for which a sufficiently large *E*_cutoff_ of 500–600 eV can be used without this correction. The correction is defined as

6 $$\Delta \mathrm{cutoff}=\Delta E_{\mathrm{low‐k},\;\mathrm{high‐E}}-\Delta E_{\mathrm{low‐k},\;\mathrm{low‐E}}$$

The Δcutoff is the difference in the calculated energy change Δ*E* (e.g., reaction energy) obtained using low and high *E*_cutoff_ (low-E and high-E, respectively) with a coarse k-point grid (low-k). The correction is added to Δ*E*_high‑k, low‑E_ to obtain an accurate energy Δ*E*_high‑k, high‑E_ at a dense k-point grid (high-k),

7 $$\Delta E_{\mathrm{high‐k},\;\mathrm{high‐E}}\approx \Delta E_{\;\mathrm{high‐k},\;\mathrm{low‐E}}+\Delta \mathrm{cutoff}$$

The Δcutoff has been reported to be largely independent of k-point meshes and can recover Δ*E*_high‑k, high‑E_ almost perfectly^97–100^ (Figure S5).

For RPA calculations of molecules involved in bulk solid and surface datasets, we extrapolated *E*_optHXX_ and *E*_c,RPA_ with respect to cell volumes (*V*) to the limit of infinitely dilute gas, using the form *E* = *E*_∞_ + α*V* ^–1^ + β*V* ^–2^, where *E*_∞_ is the energy of a molecule in an infinitely large cell^101^ (Figures S13 and S14). *E*_∞_ is correlated with the total number of electrons (*N*) and the number of valence electrons (*N*_v_) in gas species (Figure S15). Therefore, the volume dependence of molecular energies should be considered for gas species with a large number of electrons, such as I and CH_3_I, to obtain accurate adsorption energies. For DFT calculations, a large cubic cell (20 Å) was used without applying the extrapolation scheme. The energy difference between HSE06 results using 15 Å and 20 Å cells was less than 0.001 eV. The Coulomb divergence of exact exchange was corrected using the probe-charge method^102,103^ (HFRCUT=0 in VASP, which is the default), except for bulk calculations used for surface energy evaluations (see Section S2.4 of the SI).

More computational details can be found in Sections S2 and S5 of the SI. Representative input files and example calculations from each class of benchmark data are also provided in the SI.

### 2.3 Adsorption Energy Definitions

Evaluating the thermochemistry of adsorbates is nontrivial, since both gas-phase and surface properties are involved, and it is not always straightforward to establish whether error occurs due to the gas-phase reference state or the adsorbed state. Several definitions of adsorption energies exist in the literature,^104^ and here we adopt the following three definitions:

8 $$E_{\mathrm{ads}}=E_{\mathrm{slab}+\mathrm{ads}}-E_{\mathrm{slab}}-E_{\mathrm{ads}(g)}$$

9 $$E_{\mathrm{diss}}=\underset{i}{\sum }\nu_{i}\left(E_{\mathrm{slab}+\mathrm{ads},i}-E_{\mathrm{slab}}\right)-E_{\mathrm{mol}}$$

10 $$E_{\mathrm{ads},\mathrm{LS}}=E_{\mathrm{slab}+\mathrm{ads}}-E_{\mathrm{slab}}-\underset{k}{\sum }n_{k}\mu_{\mathrm{LS},k}$$

where *E*_*slab*+*ads*_ is the combined slab and adsorbate system, *E*_*slab*_ is the bare slab, *E*_*ads*(*g*)_ is the adsorbate directly in the gas-phase, *E*_*mol*_ is a gas-phase molecule that dissociates into *E*_*slab*+*ads*,*i*_, ν_*i*_ denotes the stoichiometric coefficient of adsorbate *i* in dissociative adsorption, *n*_*k*_ is the number of elemental species *k* in the adsorbate, and μ_*LS*,*k*_ is the chemical potential of an elemental species, as obtained through least-squares regression.^104^

The first definition is the most straightforward, as it represents nondissociative adsorption. However, significant errors can be introduced because the gas-phase analogues of the adsorbates may be unstable open-shell radicals with complex electronic structure not well captured by DFT (e.g., bare O atoms, CH fragments, etc.). We obtained the reference values for the first definition by applying the same approach as in ref 79 to ADS41, which used the second definition, but using different molecular energies obtained from the AtCT database^105^ and zero point energies (ZPE) from literature.^106–109^ For adsorption of CH and CH_3_, ZPE-corrected experimental values were used.^110,111^

The second definition minimizes the use of open-shell reference states by treating the adsorption event as a dissociation of a stable gas-phase species, and normalizing the energy to be per mole of adsorbate (*E*_*diss*_(*H*) = Δ*E*/2, considering H_2_ → 2H). One advantage of referencing *E*_ads_ to molecules is that spin–orbit coupling (SOC) effects are diminished. For example, the I atom has a spin–orbit stabilization energy of −0.31 eV, whereas the value for the I_2_ molecule is −0.09 eV.^112^ Given that SOC effects are not accounted for in the methods investigated here, we applied SOC correction terms to all calculated energies involving I-containing species in both adsorption definitions. Still, this approach does not guarantee reduced gas-phase error because some molecules are difficult to describe accurately using DFT, such as O_2_ (whose triplet state is the ground state). In cases where the adsorbate does not dissociate (e.g., CO, NO, CH_4_, etc.), the two definitions are identical. For other species, conversion between the definitions requires knowledge of the gas-phase molecular energies. For the *PBE+D3/M06* method, only the first definition (*E*_*ads*_) was reported in the original paper, and gas-phase energies were not reported. Therefore, we calculated molecular energies at the same level of theory, using ORCA version 5.0.4 ^113^ and the def2-QZVPP basis set to convert between the two:^114^

$$E_{\mathrm{gas}}^{PBE+D3/M06}=E_{\mathrm{gas}}^{\mathrm{PBE}+D3,\mathrm{periodic}}+\left(E_{\mathrm{gas}}^{M06,\mathrm{cluster}}-E_{\mathrm{gas}}^{\mathrm{PBE}+D3,\mathrm{cluster}}\right)$$

where *E*_gas_ includes both *E*_ads(g)_ and *E*_mol_, $E_{\mathrm{gas}}^{\mathrm{PBE}+D3,\mathrm{periodic}}$ is molecular energies at PBE+D3 level under periodic boundary conditions (calculated using VASP), and $E_{\mathrm{gas}}^{M06,\mathrm{cluster}}$ and $E_{\mathrm{gas}}^{\mathrm{PBE}+D3,\mathrm{cluster}}$ are the corresponding energies computed in a cluster model at M06 and PBE+D3 levels (calculated using ORCA).

The third definition of adsorption energies, *E*_ads,LS_, avoids issues of reference state error by obtaining reference chemical potentials using least-squares (LS) regression. The μ_LS,_ *_i_* are determined by minimizing the squared magnitude of the adsorption energy *E*_*i*_:^104^

11 $$\mu_{\mathrm{LS}}=\underset{\mu }{\arg\min}\underset{i=1}{\sum }{\left(E_{i}-\sum_{k=1}n_{i,k}\mu_{k}\right)}^{2}$$

where **μ** is a (*k* × 1) vector of elemental chemical potentials μ_*k*_, *E*_*i*_ is QM energy of species *i*, and *n*_*i*,*k*_ is the number of element *k* in species *i* (and includes only elements in the adsorbates). In this case, *E*_*i*_ corresponds to (*E*_slab+ads_ – *E*_slab_) for computed adsorption energies and (*E*_ads_ + Δ*E*_f,ads(g)_) for experimental adsorption energies, where Δ*E*_f,ads(g)_ is the formation energy of gas-phase adsorbate (i.e., the formation enthalpy at 0 K with ZPE contributions removed) obtained from AtCT. This minimization can be efficiently solved using linear algebra:

12 $$\mu_{\mathrm{LS}}={\left(N^{T}N\right)}^{-1}N^{T}E$$

13 $$\mu_{\mathrm{LS}}=\left(\begin{array}{l} \mu_{\mathrm{LS},1}\\ \mu_{\mathrm{LS},2}\\ \vdots \\ \mu_{\mathrm{LS},k} \end{array}\right),N=\left(\begin{array}{llll} n_{1,1} & n_{1,2} & \cdots & n_{1,k}\\ n_{2,1} & n_{2,2} & \cdots & n_{2,k}\\ \vdots & \vdots & \ddots & \vdots \\ n_{i,1} & n_{i,2} & \cdots & n_{i,k} \end{array}\right),E=\left(\begin{array}{l} E_{1}\\ E_{2}\\ \vdots \\ E_{i} \end{array}\right)$$

where **N** is a stoichiometry matrix (*i* × *k*) with elements *n*_*i*,*k*_ and ***E*** is an (*i* × 1) vector of QM energies *E*_*i*_ of each species. This approach eliminates the need for any gas-phase reference and can be shown to minimize the squared errors between energies from different sources.^104^ However, it introduces one degree of freedom for each element involved and can thus underestimate the error for datasets with few examples of an element (e.g., iodine). It also removes all systematic error by construction, so that systematic over- or under-binding of molecules will not show up in this definition since the error will be pushed into the reference states. Finally, the specific values of μ_LS,_ *_i_* are also dependent on the dataset used, and it is thus not a physically meaningful quantity in general. Nonetheless, it provides a useful perspective on the adsorption error since it directly probes the error that arises from the surface and adsorbate and establishes a lower bound on a practical adsorption energy error.

## 3 Results

The performance of the proposed optRPA26 method was benchmarked against the various datasets introduced earlier. For comparison, we also evaluated representative DFT functionals (PBE+D3, r2SCAN+rVV10, and HSE06+D3) and included data from higher-level methods from the literature where available. We report energy errors using mean absolute deviation (MAD), root-mean-square deviation (RMSD), and maximum absolute deviation.

For lattice constants and bulk moduli, mean absolute percentage deviation (MAPD), root-mean-square percentage deviation (RMSPD), and maximum absolute percentage deviation are reported. The reported RPA results correspond to RPA@PBE.

### 3.1 Molecular Datasets

Benchmarking results for different molecular datasets are presented in Figure 1. For W4-11 (atomization energy), PBE+D3 showed a significantly higher RMSD (0.81 eV) than other DFT functionals, but performed comparably for W4-11-RE (reaction energy) with an RMSD of 0.27 eV, suggesting substantial error cancellation. Similar behavior was observed for RPA, with RMSDs of 0.86 eV (W4-11) and 0.20 eV (W4-11-RE). The largest errors of RPA in W4-11-RE were associated with the HS molecule, and the comparable performance of RPA and PBE+D3 on gas-phase properties is consistent with prior benchmarking for ethane dehydrogenation.^115^ For BH76 (barrier height), the accuracy of DFT functionals followed their hierarchy on Jacob’s ladder, with RMSDs of 0.46 eV (PBE+D3), 0.35 eV (r2SCAN+rVV10), and 0.22 eV (HSE06+D3). Similar trends were observed for BH76RC (reaction energy) and MOBH29 (barrier height and reaction energy of TM complex), albeit with smaller differences among the functionals. DFT methods showed mutually similar accuracy for S19 (noncovalent binding energy), with RMSDs of 0.03–0.04 eV. The optRPA26 method consistently outperformed all tested methods, yielding RMSDs of 0.17 eV (W4-11), 0.06 eV (W4-11-RE), 0.09 eV (BH76), 0.10 eV (BH76RC), 0.09 eV (MOBH29), and 0.01 eV (S19). Notably, the maximum error for optRPA26 on W4-11-RE is 0.27 eV, which is substantially lower than all other methods (∼1 eV), indicating that the approach will be generally reliable for reaction energies, with similar trends observed for barrier heights. To probe known RPA failure modes beyond the benchmark datasets, the SI includes bond-dissociation curves for H_2_, N_2_, and H_2_^+^, compared against accurate reference curves from the literature.^116–118^ optRPA26 gives similar or improved behavior near equilibrium for H_2_ and N_2_, but is less accurate at larger bond distances; for H_2_^+^, it reduces the error relative to RPA@PBE, although both RPA-based curves remain qualitatively inaccurate.

**Figure 1:**
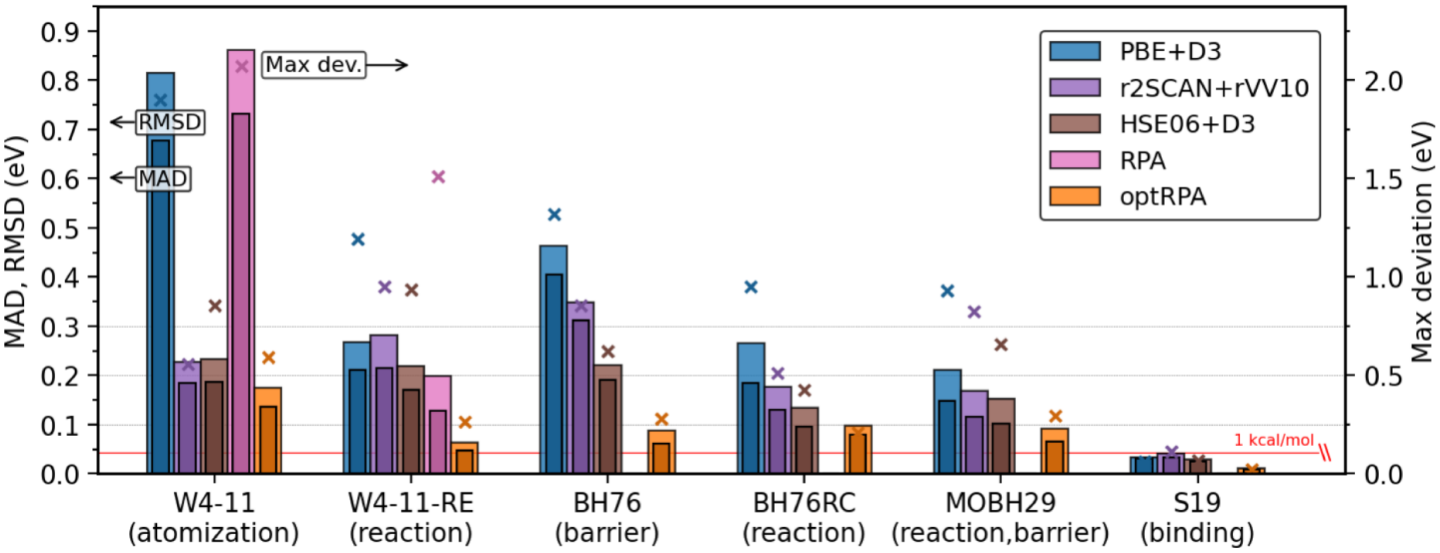
Absolute deviations from reference values for each molecular dataset. Datasets include W4-11 (*n* = 124*),^61^ W4-11-RE (*n* = 8,868*),^64^ BH76 (*n* = 76),^65–67^ BH76RC (*n* = 30),^67^ MOBH29 (*n* = 87),^68–71^ and S19 (*n* = 19).^72^ Bars represent MAD (dark color) and RMSD (light color), and × symbols indicate maximum deviations. A horizontal red line denotes the chemical accuracy of 1 kcal/mol. *W4-11 and W4-11-RE contain only the molecules listed in TAE_nonMR124.

### 3.2 Bulk Solid Datasets

#### 3.2.1 Lattice Constant and Bulk Modulus

The calculated bulk lattice constants and bulk moduli for nonoxide solids are presented in Figure 2. In both lattice constants and bulk moduli, optRPA26 showed good accuracy with RMSPD of 0.37% and 5.16%, respectively, comparable to the best-performing methods, RPA (0.48% and 4.64%) and rALDA (0.75% and 5.39%). The accuracy of rAPBE is difficult to assess because only four data points are available (for lattice constants only). PBE+D3 delivered good performance for lattice constants (RMSPD of 0.85%) and bulk moduli (8.83%), but the D3 correction was necessary because, without it, RMSPD increased to 1.64% and 14.51%, respectively. Among DFT methods, r2SCAN+rVV10 produced the most accurate results, outperforming HSE06+D3.

**Figure 2:**
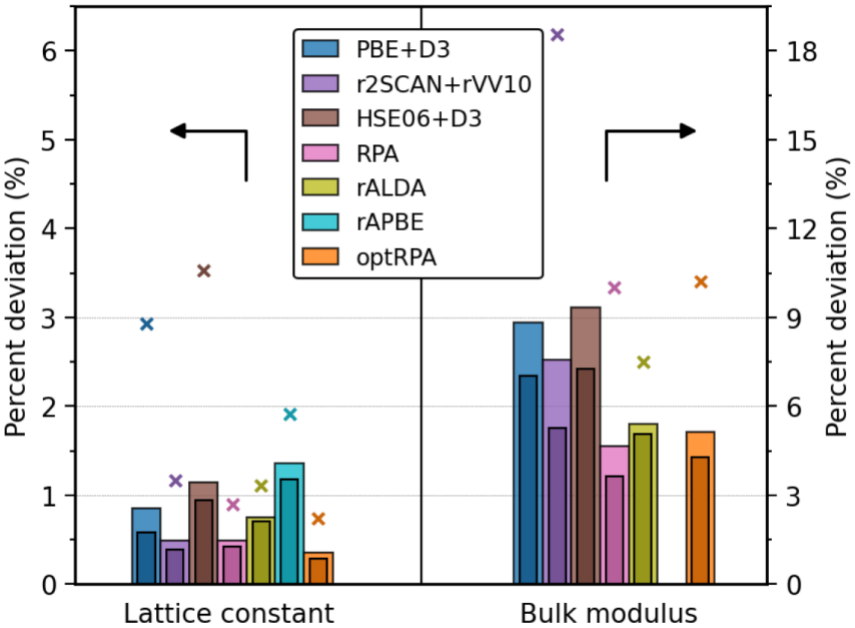
Absolute percentage deviations from ZPE-corrected experimental values for lattice constants^73^ and bulk moduli^73,74^ for 24 (nonoxide) bulk solids. RPA (*n* = 24),^73^ rALDA (*n* = 10),^119^ and rAPBE (*n* = 4)^54^ values are taken from the literature. Bars represent MAPD (dark color) and RMSPD (light color), and × symbols indicate maximum percentage deviations.

#### 3.2.2 Atomization Energy of Nonoxide and Oxide Formation Energy

The calculated atomization energies of nonoxides and oxide formation energies are presented in Figure 3. optRPA26 performed well for both datasets with RMSDs of 0.10 eV (atomization energy) and 0.12 eV (oxide formation energy), comparable to the best-performing methods for each dataset, which are rAPBE (0.07 eV) for atomization energy and r2SCAN+rVV10 (0.13 eV) for oxide formation energy, respectively. As observed earlier for lattice constants and bulk moduli, r2SCAN+rVV10 again showed the highest accuracy for the bulk properties among DFT methods. The reference values for oxide formation are not ZPE-corrected, but this effect is reported to change the MAD of the current dataset by, at most, 0.01 eV.^76^

**Figure 3:**
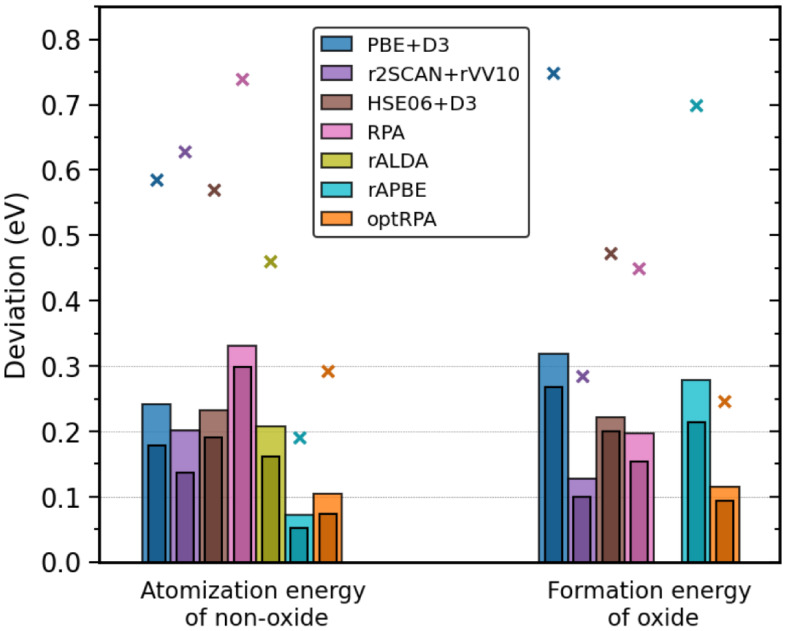
Absolute deviations from (ZPE-corrected) experimental atomization energies (eV per formula unit) of 24 nonoxides^73^ and (non-ZPE-corrected) formation energies (eV per oxygen) of 23 oxides.^76,77^ For atomization energies, RPA values (*n* = 24) are from ref 73, and rALDA (*n* = 19) and rAPBE (*n* = 19) are from ref 54. For oxide formation energies, RPA values (*n* = 22) are from ref 76, and rAPBE values (*n* = 21) are from ref 97. Bars represent MAD (dark color) and RMSD (light color), and × symbols indicate maximum deviations.

#### 3.2.3 Magnetic Moments of Metals

We evaluated the magnetic moments of elemental ferromagnets (Fe, Co, and Ni) to assess the performance of different functionals in capturing the correct spin polarization, as shown in Table 1. PBE values show good agreement with experimental values, while HSE06 (and hybrid functionals in general) tends to significantly overestimate the spin magnetic moments, with a similar trend observed for the meta-GGA r2SCAN. In contrast, LC-srPBEx25 reproduces the correct experimental magnetic moments, similar to PBE. We note that this property depends only on the LC-srPBEx25 method, not optRPA26 itself, but it confirms that the orbitals used in optRPA26 for spin-polarized metals have qualitatively accurate magnetic properties. For RPA calculations, obtaining the correct magnetic moment is important because the RPA correlation energy is evaluated in a nonself-consistent manner. However, a correct magnetic moment does not mean that LC-srPBEx25 yields better DFT energies.

**Table 1:** Spin Magnetic Moment (*μ*_B_) of Magnetic Bulk Solids Calculated with Different Functionals^a^

Method	Fe	Co	Ni
PBE+D3	2.115 (2.191)	1.586 (1.633)	0.654 (0.649)
r2SCAN+rVV10	2.544 (2.657)	1.734 (1.763)	0.740 (0.775)
HSE06+D3	2.748 (2.823)	1.841 (1.917)	0.873 (0.888)
LC-srPBEx25	2.057 (2.144)	1.586 (1.635)	0.625 (0.652)
Experiment^78^	2.13	1.52	0.57

a Values outside parentheses correspond to PBE+D3–optimized structures, while values in parentheses correspond to structures with experimental lattice constants.

#### 3.2.4 Relative Stability of Bulk Solids

We evaluated the relative stability of bulk polymorphs for Co, MoO_3_, and TiO_2_, where standard DFT often fails to predict the correct ground-state phase.^120–123^ optRPA26 correctly predicts the most stable phases for all systems considered, albeit by very small energy differences, as shown in Table 2. We note that it is possible that the experimentally observed ground-state phase may not have the lowest energy at 0 K, as thermal effects may play a role.^124^

**Table 2:** Relative Energy (eV) of Co, MoO_3_, and TiO_2_ Bulks in Different Phases^a^

	Co			MoO_3_		TiO_2_	
Method	hcp Co	fcc Co	bcc Co	α-MoO_3_	β-MoO_3_	rutile	anatase
PBE+D3	**0**	0.022	0.096	0	**–0.075**	0	**–0.014**
r2SCAN+rVV10	0	–0.024	**–0.037**	0	**–0.030**	0	**–0.002**
HSE06+D3	0	–0.225	**–0.267**	0	**–0.077**	0	**–0.009**
DMC^124^						0	**–0.041**
optRPA26	0 (0)	0.114 (0.106)	0.058 (0.059)	**0**	0.008	**0**	0.004
Experiment	stable			stable		stable	

a The smallest value (bold) corresponds to the most stable phase within each formula. Values outside parentheses correspond to PBE+D3–optimized structures, while values in parentheses correspond to structures with experimental lattice constants.^125^

### 3.3 Surface Datasets

#### 3.3.1 Surface Reactions on Metals

The performance of optRPA26 was benchmarked for adsorption energies on metallic surfaces using experimental values,^35,79^ as shown in Figure 4. A total of 27 surface reactions were investigated on nonmagnetic Cu(100), Cu(111), Ru(0001), Rh(100), Rh(111), Pd(100), Pd(111), Ir(111), and Pt(111) surfaces, as well as on the magnetic Ni(111) surface.

**Figure 4:**
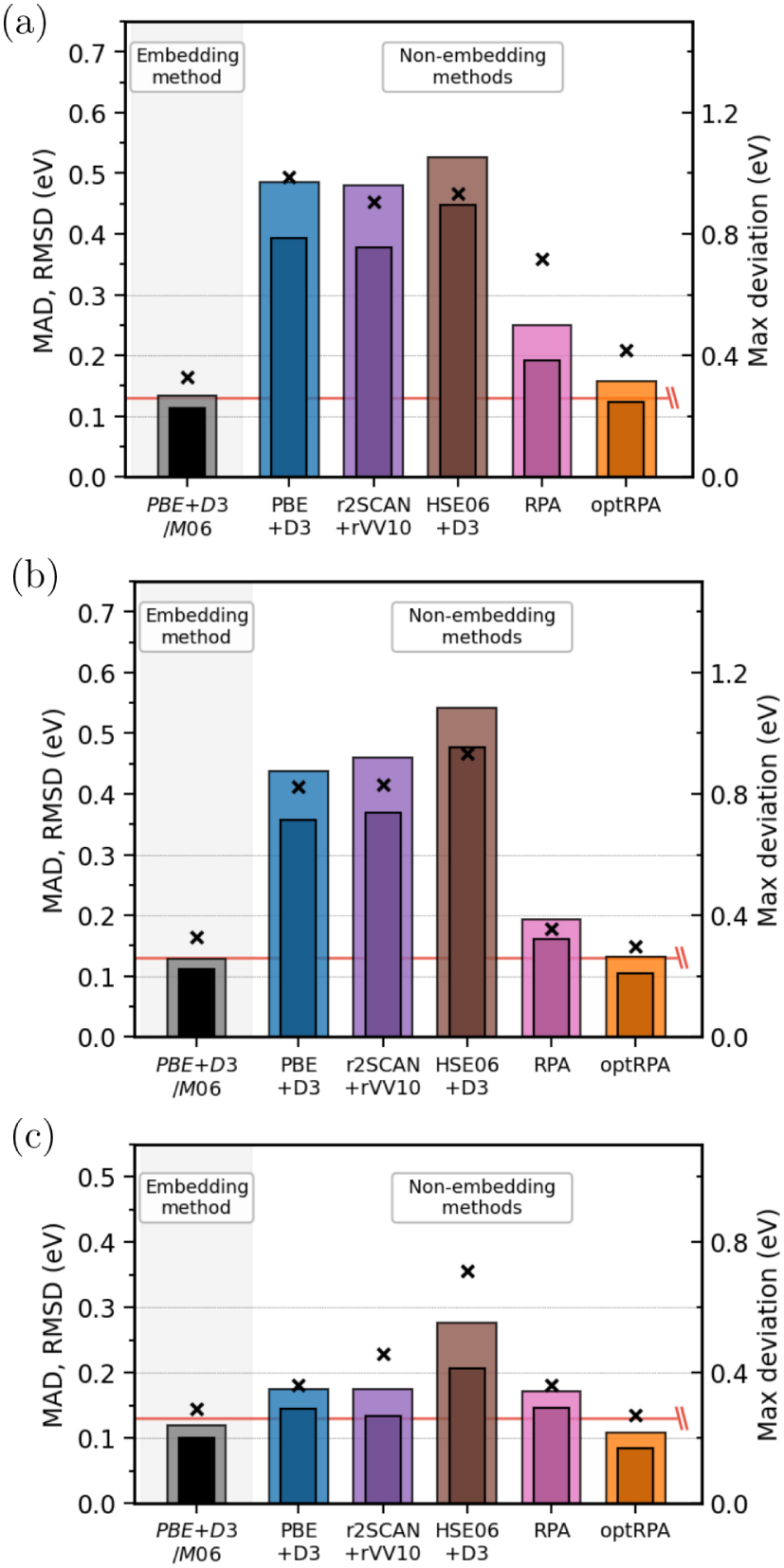
Absolute deviations from ZPE-corrected experimental adsorption energies^41^ on metals (Cu, Ru, Rh, Pd, Ir, Pt, and Ni) (*n* = 27). *PBE+D3/M06* ^41^ refers to a hybrid scheme that combines cluster (M06 level) and periodic model (PBE+D3 level). (a) Nondissociative adsorption energies referenced to gas-phase adsorbates (e.g., O) as in ref 41. (b) Adsorption energies referenced to gas-phase molecules (e.g., O_2_) as in ADS41.^35^ (c) Nondissociative adsorption energies obtained by referencing *E*(slab + adsorbate) – *E*(slab) to least-squares elemental chemical potentials, compared against experimental values adjusted by the formation energies of adsorbates. The adsorption energy values are defined on a per-adsorbate basis; for example, for O adsorption, the adsorption energy is ΔE in (a, c) or ΔE/2 in (b). Bars represent MAD (dark color) and RMSD (light color), and × symbols indicate maximum deviations. Red horizontal lines denote the transition metal chemical accuracy of 3 kcal/mol.

In Figure 4a, the reaction energies from ADS41 were recalculated to represent nondissociative adsorption energies, as defined in eq 8. We compare the results to other density functionals as in prior sections, as well as *PBE+D3/M06*. The *PBE+D3/M06* method has been reported as one of the most accurate approaches for adsorption energy calculations, showing the lowest RMSD of 0.13 eV for the current set. The optRPA26 approach achieved a comparable accuracy with an RMSD of 0.16 eV, followed by RPA (0.25 eV). Despite substantially different computational costs, various other DFT methods showed similar accuracies with RMSDs of 0.49 eV (PBE+D3), 0.48 eV (r2SCAN+rVV10), and 0.53 eV (HSE06+D3), consistent with results from ADS41.^35^ RPA performed significantly better than DFT methods, confirming its high accuracy for adsorption systems, as previously reported,^98,126^ and further supported here using a larger dataset. Despite the good performance of RPA for adsorption energies, the prior benchmarking study of ethane dehydrogenation on Pt(111) showed that RPA was not superior in predicting macroscopic observables through microkinetic modeling, compared to other DFT functionals.^115^ The reduced accuracy of these macroscopic observables is likely related to the lower accuracy of RPA for molecular energies and, in part, to the neglect of the volume dependence of molecular energies (Figure S14).

In Figure 4b, the same adsorption energy results are reported using dissociative surface reaction energies, as defined in eq 9. The results did not change significantly, and post-DFT methods showed RMSDs of 0.13 eV (*PBE+D3/M06*), 0.13 eV (optRPA26), and 0.19 eV (RPA), while standard DFT methods exhibited RMSDs of 0.44 eV (PBE+D3), 0.46 eV (r2SCAN+rVV10), and 0.54 eV (HSE06+D3). PBE+D3 and RPA values differed the most (∼0.05 eV) between the two adsorption energy definitions, reflecting their large errors in molecular atomization energies, revealing a significant conflation of gas-phase and adsorbed-state errors in adsorption energies.

To deconvolute these contributions, the least-squares adsorption energies (eq 10) are shown in Figure 4c. These adsorption energies avoid any explicit gas-phase reference by determining elemental chemical potentials by minimizing the squared magnitude of the relative reaction energies (*E*_slab+ads_ – *E*_slab_) in the dataset. The resulting elemental potentials will implicitly depend on the compositions of all adsorbed species, but do not rely on any computed gas-phase energies and are thus purely dependent on the surface–adsorbate interactions.^104^ The least-squares adsorption energies minimize bias, giving a zero mean signed error (MSD), whereas in the former two definitions, DFT methods give MSDs that are similar in magnitude to the MAD values (Figure S17), indicating systematic error in the surface–adsorbate interactions or gas-phase energies. The strong overbinding trend is reduced when excluding the dispersion correction, leading to lower RMSDs for all DFT functionals (Figure S17). The least-squares approach yields RMSDs of 0.12 eV (*PBE+D3/M06*), 0.11 eV (optRPA26), and 0.17 eV (RPA), with corresponding DFT errors of 0.18 eV (PBE+D3), 0.17 eV (r2SCAN+rVV10), and 0.28 eV (HSE06). The result is different if the least-squares approach is used to correct gas-phase energies only, in which case the gas-phase least-squares RMSDs are always lower than the RMSDs for adsorption energies, indicating that slab-adsorbate errors dominate for all methods (Table S4). Excluding the dispersion correction for DFT functionals leads to very similar RMSDs, confirming that the least-squares approach reduces the systematic bias (Table S4). Interestingly, HSE06+D3 is consistently outperformed by other DFT methods across the different referencing schemes, likely due to issues of hybrid functionals in treating metallic systems.^127,128^ The strong performance of *PBE+D3/M06* and optRPA26 across all referencing schemes reflects their low gas-phase errors, and suggests that methods maximizing error cancellation between gas-phase and adsorbed species are promising for reducing adsorption energy errors.^129,130^ However, for calculations that intrinsically involve both gas-phase and surface-mediated interactions, such as transition states of dissociative adsorption, methods like optRPA26 or *PBE+D3/M06* with inherently high gas-phase accuracy will be required.

We note a substantial difference in the adsorption energies of O and CH between our PBE+D3 values and the PBE+D3 values from ref 41. The energy offsets for O adsorption were consistent at 0.27–0.29 eV, with the values from ref 41 indicating stronger adsorption, which coincides with half of the O_2_ energy difference between the doublet and triplet states (0.33 eV). When converting adsorption energies from ref 41 to the second definition, the PBE+D3 values for O adsorption in ref 41 also show a similar energy shift relative to the PBE values in ADS41 ^35^ (beyond D3 effects) and to the PBE+D3 values for O/Pt(111) from the literature.^131,132^ A similar discrepancy was found for CH adsorption, with a larger energy difference of 0.59 eV. It is possible that these exaggerated adsorption energies may have propagated into the *PBE+D3/M06* values, and using the corrected energies could increase the error in the adsorption energy of the method by ∼0.3 eV for these adsorbates. However, these issues should not affect the least-squares results in Figure 4c, where the effects of gas-phase reference states have been removed. The similar RMSDs for *E*_ads,LS_ values at PBE+D3 level support this statement, with the values being 0.135 eV (ref 41) and 0.132 eV (our calculations). We also note a structural aspect of CH_3_I adsorption on Pt(111). In previous benchmarking studies of adsorption on metal surfaces,^34,35,41,79^ CH_3_I was modeled as adsorbing perpendicular to Pt(111). However, experimental studies have shown that CH_3_I adsorbs in a tilted configuration.^133–135^ Our DFT calculations using the tilted geometry predict that this configuration adsorbs more strongly by 0.35 eV at PBE+D3 level, compared to the perpendicular configuration. The tilted geometry is more stable with all functionals, including optRPA26, but this stronger adsorption increases error compared to experiment for functionals that already overbind CH_3_I, while it reduces the error for optRPA26.

Finally, there is a well-known trade-off between the accuracy of surface energies and adsorption energies for TM surfaces treated with standard density functionals, and RPA methods are known to perform well on both quantities.^55,136^ To evaluate whether optRPA26 is able to simultaneously capture surface and adsorption energies, we evaluated the surface energies of four TM (Cu, Rh, Pd, and Pt) surfaces with the (111) facet and plotted them against the CO adsorption energy (used in Figure 4a–b), as shown in Figure 5. The post-DFT methods generally showed good agreement with experimental values of both surface and adsorption energies of CO, and optRPA26 achieved an accuracy on surface energies comparable to or better than other post-DFT methods.

**Figure 5:**
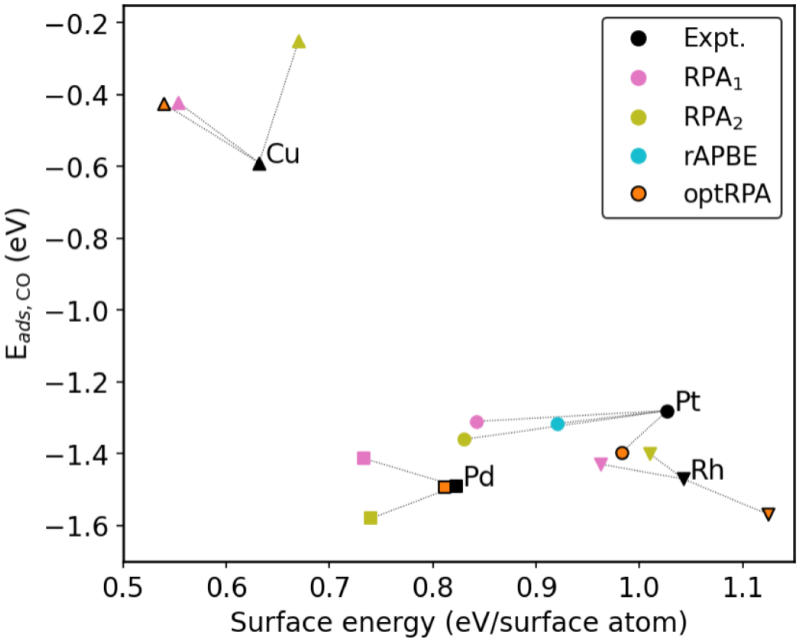
Surface energies and CO adsorption energies on four TM surfaces. The optRPA26 values for *E*_ads_ correspond to those in Figure 4. The values of RPA_1_,^136^ RPA_2_,^98^ rAPBE,^54^ and experiments^80^ are taken from the literature.

#### 3.3.2 Surface Reactions on Oxide

We evaluated the performance of optRPA26 for surface reactions on oxide surfaces using reference values from local CCSD­(T)^8,20^ or experiments when available (for nondissociative H_2_O adsorption on MgO and TiO_2_),^21^ as presented in Figure 6. The dataset includes CO adsorption energy on MgO, and H_2_O adsorption and dissociation energies and barrier heights on Al_2_O_3_ and TiO_2_ surfaces. The systems were selected because both high-level energies and corresponding structures are available. A recent study of the autoSKZCAM framework^21^ (an embedding method combining local CCSD­(T)) provides high-quality structures and energies, that could serve as a basis for further benchmarking of optRPA26 in future work.

**Figure 6:**
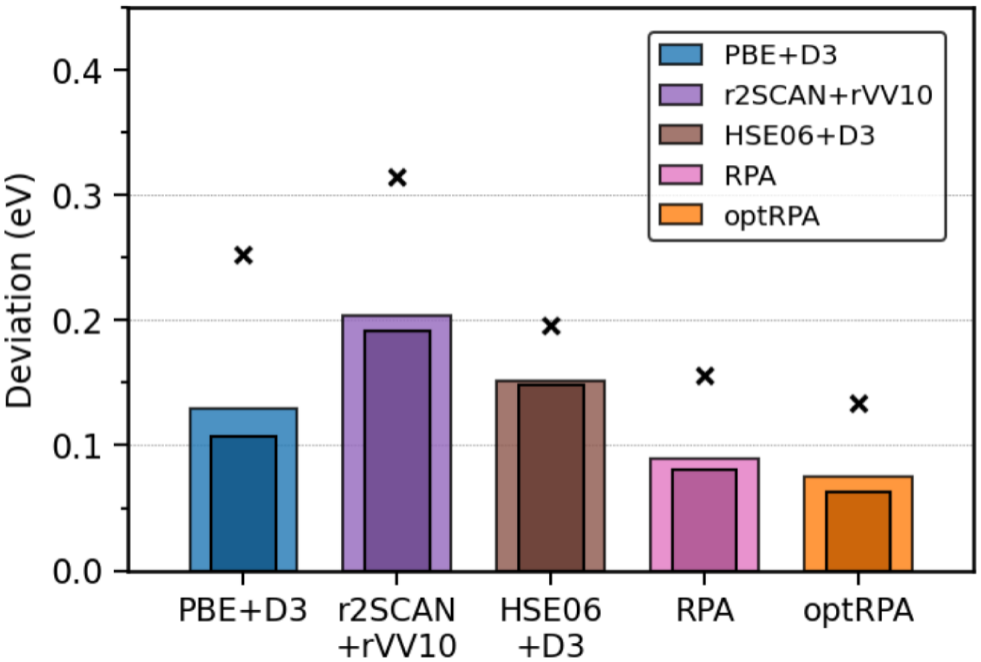
Absolute deviations from CCSD­(T) values (*n* = 5)^8,20^ or ZPE-corrected experimental values (*n* = 2) (for H_2_O adsorption on MgO and TiO_2_)^21^ for surface reaction energies on oxides (MgO, Al_2_O_3_, and TiO_2_) (*n* = 7). Bars represent MAD (dark color) and RMSD (light color), and x symbols indicate maximum deviations.

Within the set of DFT methods examined, PBE+D3 showed the lowest RMSD (0.13 eV), followed by HSE06+D3 (0.15 eV) and r2SCAN+rVV10 (0.20 eV). DFT methods significantly underestimated barrier heights for H_2_O dissociation on both the Al_2_O_3_ surface $\left(E_{a}^{\mathrm{CCSD}(T)}=0.38\mathrm{eV}\right)$ , with DFT values of 0.13 eV (PBE+D3), 0.07 eV (r2SCAN+rVV10), and 0.19 eV (HSE06+D3), and the TiO_2_ surface $\left(E_{a}^{\mathrm{CCSD}(T)}=0.32\mathrm{eV}\right)$ with DFT values of 0.26, 0.13, and 0.17 eV, respectively. The optRPA26 method showed the lowest error metrics (MAD of 0.06 eV and RMSD of 0.08 eV), but RPA performed comparably (0.08 and 0.09 eV). These RPA-based methods yield barrier heights closer to the CCSD­(T) values, namely 0.34 eV (RPA) and 0.32 eV (optRPA26) on the Al_2_O_3_ surface, and 0.46 eV (RPA) and 0.37 eV (optRPA26) on the TiO_2_ surface.

## 4 Discussion

### 4.1 Optimization of DFT Orbitals

In our previous study,^57^ we introduced an optimized RPA method (optRPA24), which used PBEx50 (a global PBE hybrid with 50% exact exchange), to calculate DFT orbitals. Although this approach worked well for gas-phase atomization and reaction energies and for nonoxide bulk properties, further testing revealed a formation energy error exceeding 0.5 eV for the TM oxide RuO_2_ (despite an RMSD of around 0.1 eV for formation energies of non-TM oxides). Moreover, the convergence of the PBEx50 wave functions was slow with respect to k-points for the adsorption energies in metals, creating a significant practical barrier to the large-scale application of optRPA24. The convergence issue is likely related to multiple local minima observed in EXX calculations of metal surfaces^25,137^ even with tight energy convergence thresholds (10^–8^ eV). In addition, the Coulomb singularity^138^ worsens the energy convergence of calculations involving EXX on metals. Although singularity correction schemes have been successfully applied to approximate the **G** = 0 contribution in DFT hybrid calculations,^127,128^ RPA is far more sensitive to changes in wave functions, making convergence particularly challenging. Finally, we found that incorporation of EXX led to quantitative and qualitative failures in the magnetic moments of magnetic metals. For example, PBE gives a magnetic moment of 2.12 μ_B_ for bulk Fe, which is close to the experimental value of 2.13 μ_B_,^78^ while hybrid functionals such as PBE0 and HSE06 predict ∼2.8 μ_B_, consistent with previous reports,^139–141^ and PBEx50 predicts the same value. Also, similar to other hybrid functionals, PBEx50 incorrectly predicts the ground-state crystal structure of Co to be BCC instead of HCP, raising further questions about its suitability for treating the electronic structure of metallic systems.

Using a lower EXX fraction can alleviate some of these issues, but does not fully resolve them. Instead, we explored a long-range corrected hybrid scheme (LC) with 25% EXX to generate orbitals for RPA evaluation. Applying 25% long-range EXX did not exaggerate the spin magnetic moment of Fe, unlike PBE0 and HSE06, which use the same EXX fraction. This behavior is consistent with the results of RSHXLDA (a long-range corrected hybrid functional with 100% EXX), where the magnetic moments of magnetic oxides were lower than PBE0 values.^142^ In addition, using long-range EXX can destroy the Fermi surface and make a metallic system nonmetallic, as observed in RSHXLDA, generating a vanishing density of states at the Fermi level in bulk Na.^142^ This behavior can be moderated by applying a smaller fraction of EXX. For example, RSHXLDA with 25% EXX retains states at the Fermi level in bulk Na, similar to LC-srPBEx25 (Figure S4). Reducing the EXX fraction also improved the energy convergence of metallic slabs with respect to k-point sampling. Nevertheless, the LC-hybrid based on the standard PBE functional required a very small range separation parameter for EXX (μ < 0.5 Å^–1^) to reproduce the correct Fe magnetic moment. A low μ limits the EXX contribution to only the very long-range region, substantially degrading the accuracy of RPA compared to non-LC approaches, which has a similar effect as decreasing the EXX fraction. The need for a low μ to reproduce the correct Fe magnetic moment may stem from the incompatibility between EXX and PBE correlation.^143^ To avoid relying on such a small μ value, we replaced PBE with the srPBE functional,^94–96^ specifically designed to handle short-range exchange and correlation contributions. This replacement enabled the reproduction of the correct Fe magnetic moment at a larger μ value of 3.0 Å^–1^, while largely preserving the RPA accuracy. However, the use of srPBE led to incorrect orbital orderings in halogen anions, most notably in F anion, where the degeneracy of the outermost *p* orbitals was broken. We attribute this qualitatively incorrect orbital description to two factors.

The first is insufficient exchange contribution, as only 25% EXX (only in the mid- to long-range region) is included to prevent energy convergence failure with respect to k-point sampling in metallic slabs. Second, range separation is handled improperly in VASP version 6.3.2 when using Libxc functionals. In a correct scheme, the sum of GGA exchange and EXX contributions should be 100%. However, in LC-srPBEx25, GGA exchange contributes only 75% at short-range (μ = 0 Å^–1^) (instead of the expected 100%), where EXX is absent (Figure S1a). We note that this issue has been partially resolved in newer versions of VASP, where an arbitrary fraction of GGA exchange can be applied, but without proper range separation for Libxc functionals (i.e., 100% GGA exchange at μ = 0 Å^–1^, but GGA exchange and EXX do not sum to 100% at μ ≠ 0 Å^–1^). The definition of LC-srPBEx25 can be specified largely in code-independent terms—the use of the Libxc srPBE functional, the long-range EXX fraction and range-separation parameter, the srPBE exchange/correlation range-separation parameter, and the srPBE exchange and correlation prefactors. In practice, the key requirement is access to the relevant Libxc functional. Because most periodic codes, including VASP, handle EXX separately and do not fully support EXX range separation through Libxc, the same Libxc-based LC-srPBEx25 definition is likely portable. Transfer to all-electron or Gaussian-basis-set packages may also introduce discrepancies from the basis set, core treatment, and range-separated implementation, so direct cross-code testing is an important future direction.

We addressed this issue of qualitatively incorrect orbital description by increasing the srPBE correlation contribution to 500% (Figure S1b), which empirically improves the balance of exchange and correlation within the present implementation (Figure S2). Moreover, this increase in correlation energy improved both the orbital ordering and Fe magnetic moment. The xc energy and the expectation value of xc potential of LC-srPBEx25 (75% x and 500% c) are much larger in magnitude than those of srPBE (100% x and 100% c) or a modified version of LC-srPBEx25 with 100% srPBE correlation, and are instead closer to PBE and HSE06 values (Figure S2a). Although this approach of exaggerating the correlation is highly empirical and not physically justified, it fully leverages the existing implementation in VASP, one of the most widely used DFT codes, without requiring any modifications to its source code, ensuring that optRPA26 is practically accessible to any practitioner with access to VASP version 6.3.2 or greater. The use of a constant prefactor that breaks an exact constraint in order to improve the resulting orbital energy levels is not without precedent in functional design; for example, the HLE16 functional^144^ rescales the semilocal exchange of HCTH/407 by a constant factor of 1.25 to improve semiconductor band gaps, and its correlation by 0.5 to preserve the molecular energetics—both factors breaking the uniform-electron-gas limit that HCTH/407 otherwise satisfies. Despite these seemingly large modifications, the resulting charge densities from LC-srPBEx25 remained largely consistent with those obtained from other functionals (Table S2). In addition, H_2_O formation energies evaluated nonself-consistently with different functionals on LC-srPBEx25 densities and orbitals were very similar to those obtained using their own self-consistent densities and orbitals (Table S3). The total energies showed similarly small differences, suggesting that the functional is physically reasonable. Although LC-srPBEx25 is highly empirical and largely ad hoc, it may help guide the development of more rigorous xc functionals with sophisticated range-separation strategies for generating RPA wave functions. We also re-emphasize that only the wave functions from the LC-srPBEx25 functional are used in optRPA26, and all energies reported ultimately use a combination of standard exact exchange energy, RPA correlation, and short-range PBE correlation as described in the Methods section.

The choice of reference orbitals also affects the magnitude of the RPA correlation energy and therefore the optimal value of the scaling constant *r*_*c*_. The scaling constant *r*_*c*_ for *E*_c,RPA_ correlates with the band gap (*E*_*g*_) between the highest occupied molecular orbital (HOMO) and the lowest unoccupied molecular orbital (LUMO).^145^ As more EXX is included, the HOMO–LUMO gap increases, leading to a larger optimal *r*_*c*_ value for compensation. Therefore, LC-srPBEx25 orbitals lead to the optimal *r*_*c*_ value of 1.114 when the exchange component of the total energy is evaluated using HXX. In optRPA24, PBEx50 orbitals lead to the optimal *r*_*c*_ value of 1.18. This trend is related to the dependence of *E*_c,RPA_ on the independent-particle response function χ^0^(*i*ω) and on *E*_*g*_.

$$E_{c,\mathrm{RPA}}=\int_{0}^{\infty }\frac{d\omega }{2\pi }\mathrm{Tr}\left[\ln\left(1-\chi^{0}\left(i\omega \right)v\right)+\chi^{0}\left(i\omega \right)v\right]$$

Here, χ^0^(*i*ω) is the independent-particle response function evaluated on the imaginary-frequency axis, and *v* is the Coulomb kernel. The trace is evaluated in reciprocal space, as discussed in ref 45.

$$\chi^{0}\left(i\omega \right)\propto \underset{n,n^{\prime }}{\sum }\frac{W_{nn^{\prime }}}{\varepsilon_{n^{\prime }}-\varepsilon_{n}-i\omega }$$

Here, *n* and *n*′ denote occupied and unoccupied states, respectively, $W_{nn^{\prime }}$ collects the occupation-factor and transition-matrix-element terms, and *ε* denotes band energies. A larger *E*_*g*_ therefore generally suppresses the magnitude of χ^0^(*i*ω), and reduces the magnitude of *E*_c,RPA_, requiring a larger *r*_*c*_ to compensate.

### 4.2 Optimization of Exchange and Correlation Parts of optRPA26

The scaling constant for the RPA correlation in optRPA26 was optimized by fitting to W4-11.^61^ For this procedure, we used the W4-11 values provided in GMTKN55,^146^ which are zero-point exclusive, clamped-nuclei, nonrelativistic (i.e., no corrections for scalar relativistic and spin–orbit coupling effects), core–valence-corrected, designed for benchmarking of DFT functionals. However, PAW pseudopotentials in VASP are scalar relativistic and use the frozen-core approximation.^81^ To make our results consistent with the reference values, we subtracted the scalar relativistic correction and added core–valence correction to our calculated values, leaving the original reference values unchanged. The same procedure was applied to W4-11-RE.^64^ To improve numerical stability and magnetic properties in metallic surfaces, the non-RPA correlation part of the total energy is defined using optHXX, which combines 100% EXX and 20% srPBE correlation (with μ_*c*_ = 1.89 Å^–1^ = 1.0 Bohr^–1^). This construction is motivated by the observation that energy convergence with respect to k-points is more easily achieved when evaluating the total energy directly, rather than treating the non-RPA correlation term and RPA correlation term separately. In metallic slabs, the non-RPA correlation and RPA correlation energies tend to be highly correlated. As a result, scaling the RPA correlation energy alone—especially with large *r*_*c*_—worsens the energy convergence (Figure S8). Moreover, using a large *r*_*c*_ can result in physically unreasonable behavior in magnetic systems, where the spin-polarized state has a higher total energy (i.e., is more unstable) than the spin-paired state. The range separation parameter μ_*c*_ and srPBE correlation fraction were tuned to minimize *r*_*c*_ while preserving most of the accuracy on the W4-11 and W4-11-RE datasets. Different choices of these two parameters did not lead to a significant change in accuracy as long as a similar *r*_*c*_ was obtained. Defining the non-RPA correlation part through optHXX (obtained by applying 20% srPBE correlation with μ_*c*_ = 1.0 Bohr in the HXX calculation) reduces the optimized scaling constant from 1.114 to 1.02, indicating that the underestimation of *E*_c,RPA_ can be largely compensated by incorporating a fraction of GGA correlation. Alternatively, adding a fraction of srPBE or PBE correlation energy (*E*_c,(sr)PBE_) (or subtracting −*V*_c,(sr)PBE_ + *E*_c,(sr)PBE_, which is printed in the VASP output) to HXX yields similar results. We note that inclusion of short-range PBE correlation is conceptually similar to the RPA+ approach,^48,49^ although our results were similar even if full range PBE correlation is used. Taken together, these results demonstrate transferability of the fixed LC-srPBEx25/optRPA26 construction across the tested molecular, bulk, and surface systems. Including the strongly multireference molecules excluded from fitting raises the W4-11 atomization-energy RMSD only modestly, from 0.17 to 0.20 eV, although such systems remain outside the primary validated regime. A more systematic, nonempirical determination of these parameters would be desirable; dielectric-dependent hybrid functionals provide one such example.^147,148^ In these functionals, the exact-exchange fraction is fixed by the macroscopic dielectric constant. Such prescriptions are, however, defined most naturally for bulk solids: the macroscopic dielectric constant is not well-defined for isolated molecules or slab models, and computing this quantity adds a system-dependent cost. A nonempirical construction applicable uniformly to molecules, solids, and surfaces therefore remains an open direction for future work.

### 4.3 Numerical Convergence, Physical Accuracy, and Computational Cost

In standard DFT approaches it is straightforward to perform convergence testing to control numerical error associated with k-point sampling and planewave cutoff. However, these issues demand more attention when using RPA-based approaches. Despite the significantly improved k-point convergence of adsorption energies on metals with optRPA26, residual RMSDs (relative to k60 values, where a k60 mesh corresponds to a k-point grid of [round­(60/x), round­(60/y), 1]) of less than 0.1 eV were still observed for k35–k55 meshes (Figure S7). The total energy convergence is similar to or slightly worse than that of the adsorption energies (Figure S8), and bulk systems tend to be more stable (Figure S9). Magnetic slabs and 3 × 3 slabs exhibit poorer total energy convergence behavior, but the adsorption energy starts to converge around k40–k45. As noted earlier, this likely originates from difficulties in k-point sampling for EXX calculations involving metals with highly non–analytic Brillouin–zone integrals, where a more accurate description requires statistical sampling of k-points, for example, through twist averaging,^137,149^ which is computationally much more demanding. A more approximate k-point sampling (i.e., averaging adsorption energies over three consecutive k-mesh scales) also helps achieve energy convergence using only coarse k-point grids (Figure S10). When computing RPA-based adsorption energies on metals, it is critical to carefully check k-point convergence and increase k-point density beyond what is typically needed for semilocal calculations; all results in the main text use a k50 mesh, which is much higher than the k20–k30 meshes typical with semilocal functionals. Conversely, oxide surfaces show significantly faster energy convergence, making it straightforward to include additional system-size correction terms, such as surface thickness and supercell size.

We also note that, when comparing to experimental values, there are other sources of uncertainty beyond the electronic structure. Uncertainty in experimental measurements for adsorption energies is typically on the order of 0.05 eV, with an average reported uncertainty of 0.07 eV.^41^ Furthermore, physical approximations such as the precise atomic geometry of the active site and adsorbate, the number of layers in the slab, the size of the supercell and vacuum spacing, and the slab approximation itself can introduce errors that are likely also on the order of 0.05–0.1 eV. Finally, numerical approximations such as the pseudopotentials used^150,151^ and the residual errors from k-point sampling, plane wave cutoff, smearing width, and FFT grid can also introduce errors of 0.01 – 0.05 eV, or higher. Taken together, it becomes difficult to differentiate signal from noise when evaluating the slow convergence with respect to k-points, and it is likely unreasonable to expect errors below ∼0.05–0.1 eV when comparing to experimental adsorption energies on metals. The case of oxide surface reaction energies is more straightforward, since comparisons to other computational results eliminate the uncertainty around the structural model used, and the insulating nature of oxides makes k-point convergence much faster. Indeed, the errors of optRPA26 for oxide surface reaction energies are somewhat lower (∼0.08 eV), which supports that at least some of the errors in metallic adsorption energies may arise from these relatively small but uncontrolled sources of uncertainty.

It is also worth considering the computational cost of optRPA26 as compared to other commonly used methods. In general, optRPA26 calculations consist of three parts: (1) optimization of occupied bands in LC-srPBEx25 wave functions (via a self–consistent field (SCF) cycle), (2) one-step determination of the unoccupied bands by exact diagonalization of the KS Hamiltonian using the previous wave function, and (3) evaluation of the ACFDT-RPA correlation energy. The computational cost of optRPA26 is highest for metal adsorption systems. For LC-srPBEx25 calculations of a typical metal slab, one SCF iteration within an SCF cycle takes 20–100 CPU hours at k50 mesh with *E*_cutoff_ = 300 eV, whereas the cost is negligible for PBE calculations (0.1–0.2 CPU hours). The high cost of LC-srPBEx25 is driven by the exact exchange calculation. HSE06 has an even higher computational cost of 50–300 CPU hours per SCF iteration because a larger *E*_cutoff_ of 600 eV was used, compared to 300 eV for LC-srPBEx25. Naturally, exact timings and performance will vary depending on computing resources, but in general the step that requires the most CPU time in optRPA26 is the SCF cycle to obtain the LC-srPBEx25 wave funtions, while the step that requires the most memory is evaluating the RPA correlation energy. Detailed examples of how to run optRPA26 calculations with VASP, along with additional notes on balancing computational cost with accuracy, are provided in the SI.

## 5 Conclusion

We have improved the previously reported optRPA24 method to make it applicable to general systems, including magnetic metal surfaces. This improvement was achieved by fine-tuning the underlying xc functional to produce orbitals with correct non-energy properties, such as magnetic moments and orbital orderings, while also ensuring energy convergence with respect to k-point sampling in metallic systems. This is important because the RPA correlation energy is evaluated nonself-consistently on the reference orbitals, so qualitatively incorrect electronic configurations lead to incorrect RPA energies. In benchmarking across diverse systems, optRPA26 showed high and well-balanced accuracy, with RMSDs of 0.06–0.16 eV. The accuracy for chemisorption on metal surfaces is particularly noteworthy, since the approach is competitive with the most accurate PBE+D3/M06 embedding method reported. This high accuracy and broad applicability for chemisorption on metals and other diverse properties demonstrate the capability of optRPA26 to be used as a reference method, especially when accurate experimental data is not available and CCSD­(T)-level calculations are not feasible. This study also highlights the potential of optimized RPA and related double-hybrid approaches for achieving higher accuracy than standard RPA, and suggests that further work in the development of specialized functionals for creating wave functions for RPA correlation calculations could enable methods that are more accurate and robust.

## Supplementary Material





## Notes

Structures used for the molecular, bulk, and surface benchmarks; three benchmark-result spreadsheets; and representative VASP calculation directories for atomic O, bulk BeO, and Pt(111). The example directories include POSCAR and OUTCAR files and result summaries, and the archive also provides run_rpa.py and calculation notes (ZIP)
Benchmark dataset details; dataset-specific computational settings; LC-srPBEx25 functional details; energy-convergence tests; DFT adsorption energies without dispersion correction; notes for RPA calculations; benchmark-result spreadsheets; example calculations (PDF)
