# Supplementary material for: Optimization of Random Phase Approximation Calculations for Improved Energies of Molecules, Solids, and Surfaces
Source: J Chem Theory Comput. 2026 Jul 30;22(15):7882–96. doi: 10.1021/acs.jctc.6c00537 (PMC13471736; doi:10.1021/acs.jctc.6c00537)
Supplement: Supplementary file 1 [file ct6c00537_si_002.pdf]

Supporting Information for

"Optimization of Random Phase Approximation Calculations for Improved Energies of Molecules, Solids, and Surfaces"

Neung-Kyung Yu,[1] Johannes Voss,[2] and Andrew J. Medford[1]

[1]School of Chemical & Biomolecular Engineering, Georgia Institute of Technology, Atlanta, Georgia 30332, USA

[2]SUNCAT Center for Interface Science and Catalysis, SLAC National Accelerator Laboratory, Menlo Park, California 94025, USA

1 Details of Benchmark Datasets

Table S1: Benchmark datasets, reference types, details, and literature sources used in this work.

Dataset	Ref. type	Details	References
**Molecular datasets**			
W4-11	Comput.	Atomization energies of small molecules and radicals; 124 nonMR systems used.	[62,63,148]
W4-11-RE	Comput.	Reaction energies derived from W4-11; 8,868 reactions selected from the nonMR subset.	[65,148]
BH76	Comput.	Molecular barrier heights.	[66–68,148]
BH76RC	Comput.	Reaction energies derived from BH76.	[68,148]
MOBH29	Comput.	Barrier heights and reaction energies of organometallic TM complexes.	[69–72]
S19 (from S66)	Comput.	19 non-covalent interaction energies selected from S66.	[73,148]
**Bulk datasets**			
Bulk non-oxides	Expt.	24 lattice constants, 24 bulk moduli, and 24 atomization energies of non-oxides.	[74,75]
Bulk oxides	Expt.	23 oxide formation energies.	[77,78]
Magnetic moments	Expt.	Magnetic moments of Fe, Co, and Ni.	[79]
Relative phase stability	Mixed	Relative stability of Co, MoO_3, and TiO_2 polymorphs.	Co,[122] MoO_3,[123,124] TiO_2[125,126]
**Surface datasets**			
Adsorption on metal	Expt.	27 adsorption energies derived from ADS41.	[34,35,80]
Oxide surface reactions	Mixed	Seven surface reaction energies on MgO, Al_2O_3, and TiO_2; five CCSD(T)-level and two experimental values.	MgO,[8] Al_2O_3 and TiO_2,[20] Expt. and ΔH[21]
Surface energies	Expt.	Surface energies of four transition-metal (111) surfaces.	[81]

* W4-11, W4-11-RE, BH76, BH76RC, and S19 (from S66) are subsets of GMTKN55,[148] and the relevant data can be found at https://www.chemie.uni-bonn.de/grimme/de/software/gmtkn/gmtkn55.

1.1 Molecular datasets

The datasets for molecules include subsets of W4-11 (molecular atomization energy)[62] and W4-11-RE (reaction energies derived from W4-11),[65] BH76 (barrier heights of molecular reactions),[66–68] BH76RC (reaction energies derived from BH76),[68] MOBH29 (barrier heights and reaction energies of transition metal (TM) complexes),[69–72] and the S19 subset of S66

(non-covalent interaction energies of molecular complexes).[73] W4-11, BH76, BH76RC, and S66 are subsets of the GMTKN55 benchmark database,[148] whose values were used as reference values in this study. Charge and multiplicity values reported in the datasets were used.

The **W4-11** dataset contains 140 atomization energies of small first- and second-row molecules and radicals.[62] The reference energies were calculated using highly accurate W4 theory, which includes post-CCSD(T) contributions.[63] Its superior accuracy, along with the fact that error cancellation is less likely in atomization energies than in reaction energies, makes it well suited for optimizing the scaling constant for RPA correlation energy. In W4-11, we only considered 124 molecules with less multireference character (TAE_nonMR124 subset of W4-11[62]), which are expected to be more representative of molecules relevant to catalysis and provide a more balanced data set for optimization, because systems with strong multireference character are more difficult to treat reliably even with high-level methods such as CCSD(T) and RPA-based approaches. The excluded molecules include C_2, FOOF, and S_4, and a complete list can be found in Ref. 64. The results for the full set can be found in the spreadsheet in the SI. The **W4-11-RE** dataset,[65] derived from W4-11, contains 11,247 reaction energies. Similarly to W4-11, we selected only reactions involving molecules in TAE_nonMR124, resulting in 8,868 reactions. The **BH76** dataset contains 76 barrier heights of hydrogen transfer, heavy atom transfer, nucleophilic substitution, unimolecular and association reactions.[66–68] The **BH76RC** dataset, derived from BH76, consists of 30 reaction energies.[68] The **MOBH29** dataset, which is a revised version of MOBH35,[69] contains 29 forward and 29 backward barrier heights, as well as 29 reaction energies of organometallic TM complexes.[69–72] MOBH29 does not contain reactions 17–20 and 24–25 from the original dataset, and reference values were obtained from Ref. 71. The S66 dataset contains 66 interaction energies of biomolecules containing H, C, N, and O elements.[73] We considered the **S19** subset, introduced in this work and derived from the original S66 set,[73] which includes 19 interaction energies. Of these, 14 data points were selected based on the 10 worst-performing

cases for each DFT functional considered in this study.

1.2 Bulk solid datasets

For bulk solids, we considered datasets containing 24 lattice constants, 24 bulk moduli, and 24 atomization energies of non-oxides,[54,74,76,121] as well as a dataset of 23 oxide formation energies.[77,78,99] For reference values of bulk moduli, zero-point energy (ZPE)-corrected values from a previous study were used.[75] The oxide formation dataset includes all data points from Ref. 77, except for LiO_2, whose most stable form was not available in the Materials Project,[93] and it includes one additional TM oxide, MoO_3.[78]

We also tested the magnetic moments of magnetic solids (Fe, Co, and Ni) and examined the relative stability of bulk phases of Co (hcp, fcc, and bcc phases), MoO_3 (α and β phases), and TiO_2 (rutile and anatase phases), as DFT (or specific functionals) fails to predict the correct ground-state phase.[122–125]

1.3 Surface datasets

We considered surface reactions involving metal and oxide surfaces, as well as surface energies of selected metals. For surface reactions on metals, we used a subset of the ADS41 dataset,[35] which includes CE39[34] and contains 41 experimental adsorption energies, and selected 27 data points for evaluation.

For surface reactions on oxide surfaces, seven reactions on MgO, Al_2O_3, and TiO_2 surfaces were examined using CCSD(T)-level reference values[8,20] and two experimental values (for non-dissociative H_2O adsorption on MgO and TiO_2).[21] Here, adsorption energies were evaluated using the second definition, with the energies referenced to a gas-phase reactant (CO or H_2O).

Surface energies were evaluated for four transition metal (Cu, Rh, Pd, and Pt) surfaces with (111) orientation. Data from experiment[81] and other RPA-based methods[55,100,138] were taken directly from literature sources.

2 Dataset-Specific Computational Details

2.1 Supplementary archive

The supplementary archive `optRPA26-SI.zip` contains the structures used for the molecular, bulk, and surface benchmarks; three benchmark-result spreadsheets; and representative VASP calculation directories for atomic O, bulk BeO, and Pt(111). The example directories include `POSCAR` and `OUTCAR` files and result summaries, and the archive also provides `run_rpa.py` and calculation notes.

2.2 Settings for molecular datasets

For the RPA calculations of molecules, a single Γ-point and E_{cutoff} of 600 eV were used, except for MOBH29. All DFT calculations used the same E_{cutoff} of 600 eV. Gaussian smearing was applied with a smearing width of 0.001 eV. The molecular structures from the original datasets were used. Each molecule was placed in a 10 Å cubic cell, except for MOBH29 and S66. Due to the large molecular sizes in MOBH29, the molecules were placed in a larger cubic cell (14.5 Å) and rotated to ensure that the minimum distance between any two atoms in adjacent periodic images was larger than 5.5 Å, with E_{cutoff} set to 500 eV for RPA calculations only. Similarly, for S66, a larger cubic cell (12 Å) was used to maintain a minimum inter-image atom–atom distance of 6 Å. For the subsets of GMTKN55 (W4-11, W4-11-RE, BH76, BH76RC, and S19), the corresponding charge and multiplicity from the original dataset were used. For MOBH29, only the charge was applied.

2.3 Settings for bulk solid datasets

Gaussian smearing was applied with a smearing width of 0.01 eV. The Γ-point-centered k-point grids were generated using a density-based scheme equivalent to `KSPACING` in VASP, defined as $\max(1, \mathrm{ceil}(2\pi \cdot |\mathbf{b}_i|/d_k))$, where $\mathbf{b}_i$ is the reciprocal lattice vector, d_k is the target k-point effective length scale (in Å^{-1}), and $\mathrm{ceil}(x)$ returns the smallest integer greater than

or equal to x. The k-point density is defined as $1/d_k$ (in Å).

For non-oxide solids, we used E_{cutoff} of 600 eV and k-point densities of 3.2 Å for insulators and 4.5 Å for metals to calculate lattice constants, bulk moduli, and atomization energies in both DFT and RPA calculations. When evaluating E_{optHXX}, the FFT grid for the exact exchange was set to "Accurate" to ensure more reliable energy derivatives for computing the bulk modulus. The reported lattice constants and bulk moduli were obtained by fitting the Birch–Murnaghan equation of state to energies computed at seven volumes centered around the experimental values (spanning 90–110%). The atomization energies were evaluated using the structures corresponding to the fitted lattice constants.

Oxide formation energies were calculated using PBE+D3-optimized structures for all systems involved. The Δcutoff was applied for the calculation of oxide formation energy. The "low-k" and "high-k" in Eqs. (6–7) correspond to k-point densities of 2.0 $Å^{-1}$ and 4.0 $Å^{-1}$ for oxides, and 3.0 $Å^{-1}$ and 6.0 $Å^{-1}$ for metals, respectively. The "low-E" value corresponds to E_{cutoff} of 400 eV, and "high-E" values are 900 eV (for exchange) and 600 eV (for correlation). DFT calculations were performed using the "high-k" and "high-E" (for exchange).

2.4 Settings for surface datasets

Unlike in bulk calculations, the k-point grids here are defined using a k-mesh scale, based on real-space supercell dimensions. For example, the k-mesh scale "k35" corresponds to a k35 mesh or a Γ-point-centered k-point grid of $[\text{round}(35/A), \text{round}(35/B), 1]$, where A and B are the x and y dimensions of the supercell and round denotes rounding to the nearest integer.

For most surface reactions on metals, the calculations employed 2×2, four-layer slabs with a vacuum spacing of 13.5 Å. The reactions involving O/Pt(111) and C_3H_8/Pt(111) were calculated using 3×3 slabs. The surface structures were optimized using PBE+D3, with the two bottom layers fixed. Gaussian smearing was applied with smearing widths of 0.1 eV (for DFT) and 0.01 eV (for RPA). The Δcutoff was applied for RPA calculations.

The "low-k" and "high-k" correspond to k35 (or k25 for 3×3 slabs) and k50 meshes. The k50 mesh corresponds to k-point densities of 6–7.5 Å. The "low-E" and "high-E" correspond to E_{cutoff} values of 300 eV and 500 eV. Results of the convergence tests can be found in Fig. S5 (E_{cutoff}), Fig. S6 (smearing width), and Fig. S7 (k-point). DFT calculations were conducted using k50 mesh and E_{cutoff} of 600 eV.

For oxide surfaces, we used structures from the literature from which the CCSD(T) reference values were obtained[8,20] and applied a vacuum spacing of 13 Å. The Δcutoff was applied for RPA calculations. The "low-k" corresponds to k1 mesh, while "high-k" corresponds to k20 (for TiO_2) and k25 meshes (for MgO and Al_2O_3). The "low-E" is 400 eV, and "high-E" values are 900 eV (for exchange) and 600 eV (for correlation). DFT calculations were performed using the "high-k" and "high-E" (for exchange). More details about the structures, as well as an additional correction term for slab thickness ($\Delta layer < 0.05$ eV) (applied to MgO and TiO_2) and for supercell size ($\Delta supercell \sim 0.01$ eV) (applied to MgO) are provided in the Oxide surface section of the SI. The results of energy convergence can be found in Fig. S11 and S12.

For metal surface energies, PBE+D3-optimized bulk and surface structures were used. The surface models consisted of 1×1 six-layer slabs, with the two middle layers fixed during structural relaxation. The Δcutoff was applied for both bulk and surface systems. For bulk calculations, the "low-k" and "high-k" correspond to k-point densities of 3.0 Å and 7.0 Å. For surface, "low-k" and "high-k" are k35 and k80 meshes. For both systems, "low-E" and "high-E" correspond to E_{cutoff} values of 300 eV and 500 eV. For accurate surface energies, highly converged bulk energies are required, since the total energy of six bulk units is subtracted from that of the slab (i.e. three bulk units per surface). Therefore, the Alavi-Spencer scheme[129] was used to correct the Coulomb divergence (`HFRCUT`=-1 in VASP) during bulk calculations.

3 LC-srPBEx25 Functional

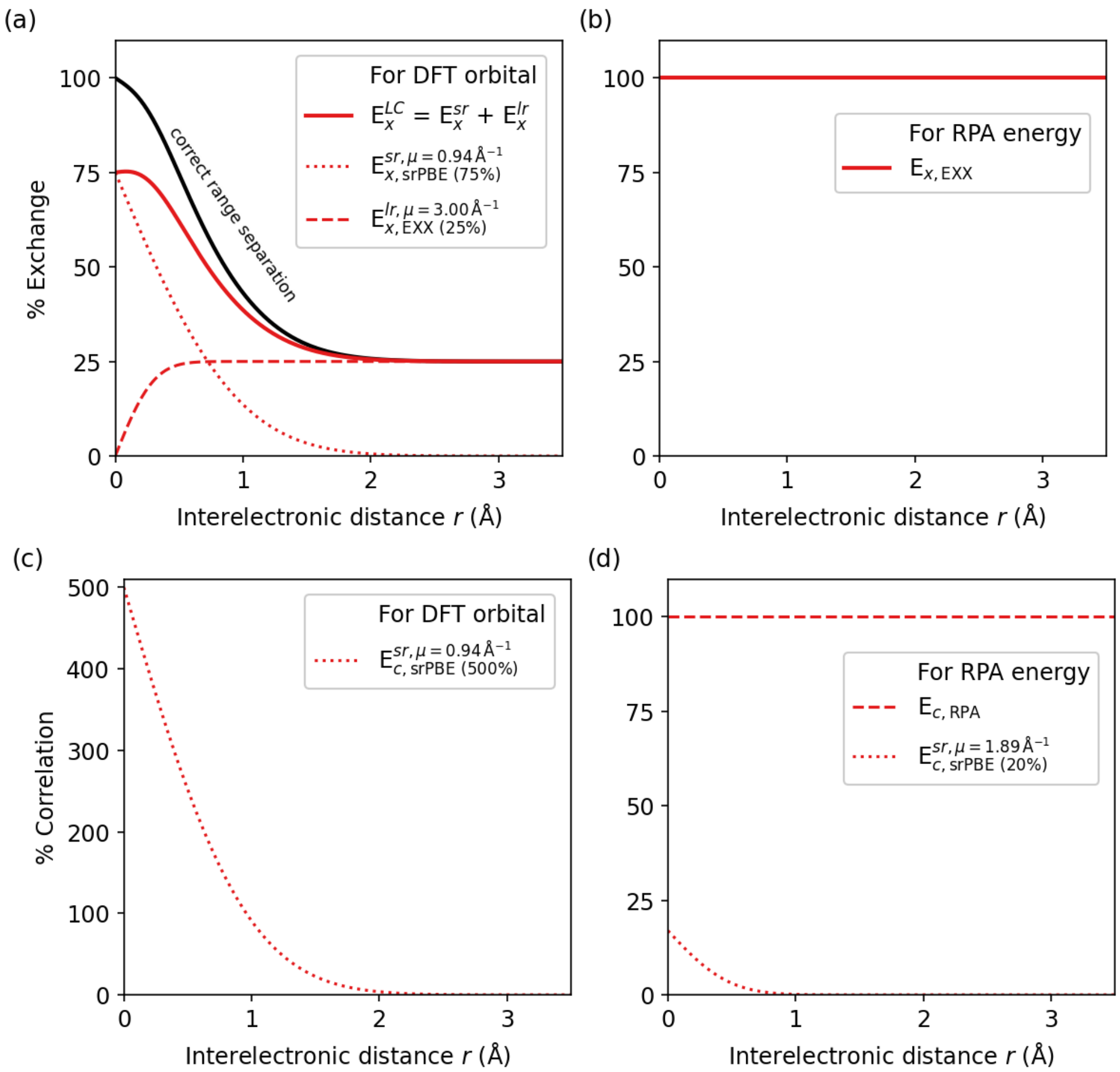

Figure S1: Range separation of exchange (a, b) and correlation (c, d) for LC-srPBEx25 functional (a, c) and the RPA energy evaluation (b, d). The incorrect range separation of exchange in LC-srPBEx25 (solid red line in (a)) is compensated by increasing the srPBE correlation to 500% (c). In (d), a small fraction of srPBE correlation is applied when evaluating the exchange part of RPA energy to reduce the scaling constant closer to 1.

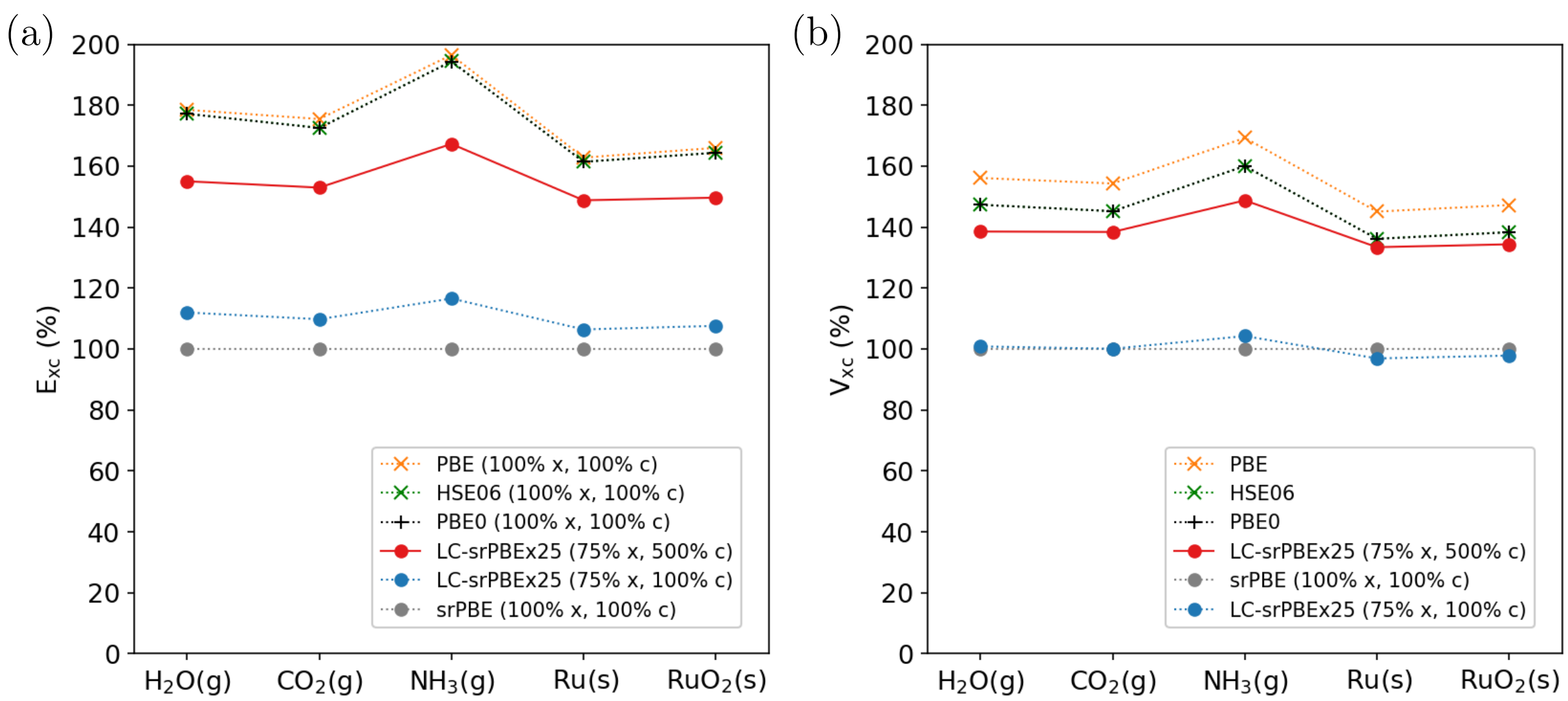

Figure S2: The xc energy (a) and the expectation value of xc potential (b), shown relative to srPBE with 100% exchange and 100% correlation, for different functionals. EXX contributions are included. Increasing the correlation contribution in LC-srPBEx25 compensates for the underestimated exchange contributions.

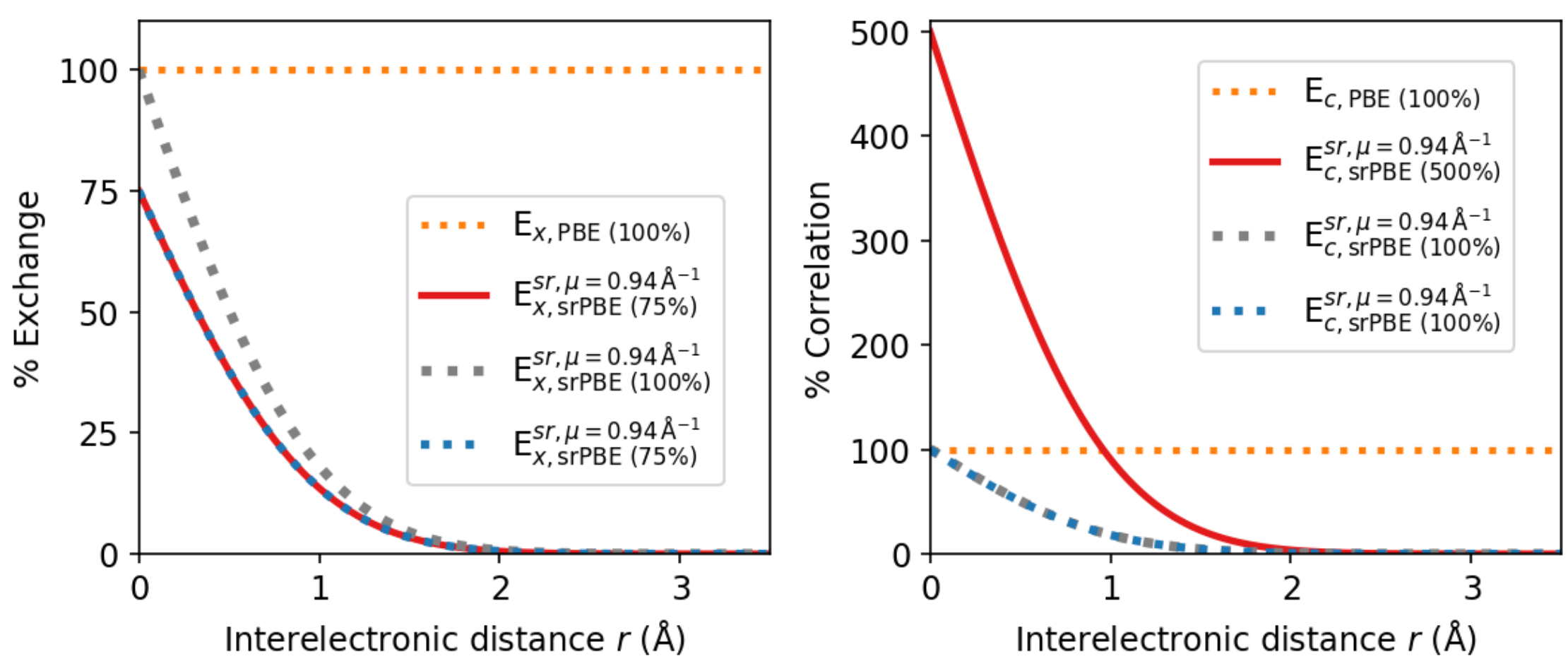

Figure S3: Range separation of exchange (left) and correlation (right) for PBE and LC-srPBEx25 functionals with different amount of xc contributions. Colors correspond to Fig. S2.

Table S2: MAD (RMSD) of charge densities relative to HSE06 values ($\times 10^{-4}$ Ry^{-3}).

**Method**	**Si bulk**	**Si atom**	**H_2O**
HSE06+D3	0	0	0
PBE+D3	2.80 (4.55)	0.05 (0.35)	0.12 (1.44)
r2SCAN+rVV10	0.84 (1.43)	0.05 (0.36)	0.03 (0.48)
LC-srPBEx25	1.98 (3.41)	0.13 (0.60)	0.24 (4.91)

Table S3: H_2O(g) formation energy (eV) evaluated using various combinations of functionals. Columns correspond to functionals used to obtain charge densities and orbitals, and rows to those used to evaluate the energies in a non-self-consistent manner. Diagonal values represent self-consistent results.

	**HSE06+D3**	**PBE+D3**	**r2SCAN+rVV10**	**LC-srPBEx25**
HSE06+D3	-2.638	-2.642	-2.642	-2.679
PBE+D3	-2.516	-2.512	-2.518	-2.531
r2SCAN+rVV10	-2.495	-2.497	-2.491	-2.551
LC-srPBEx25	-3.761	-3.735	-3.782	-3.718

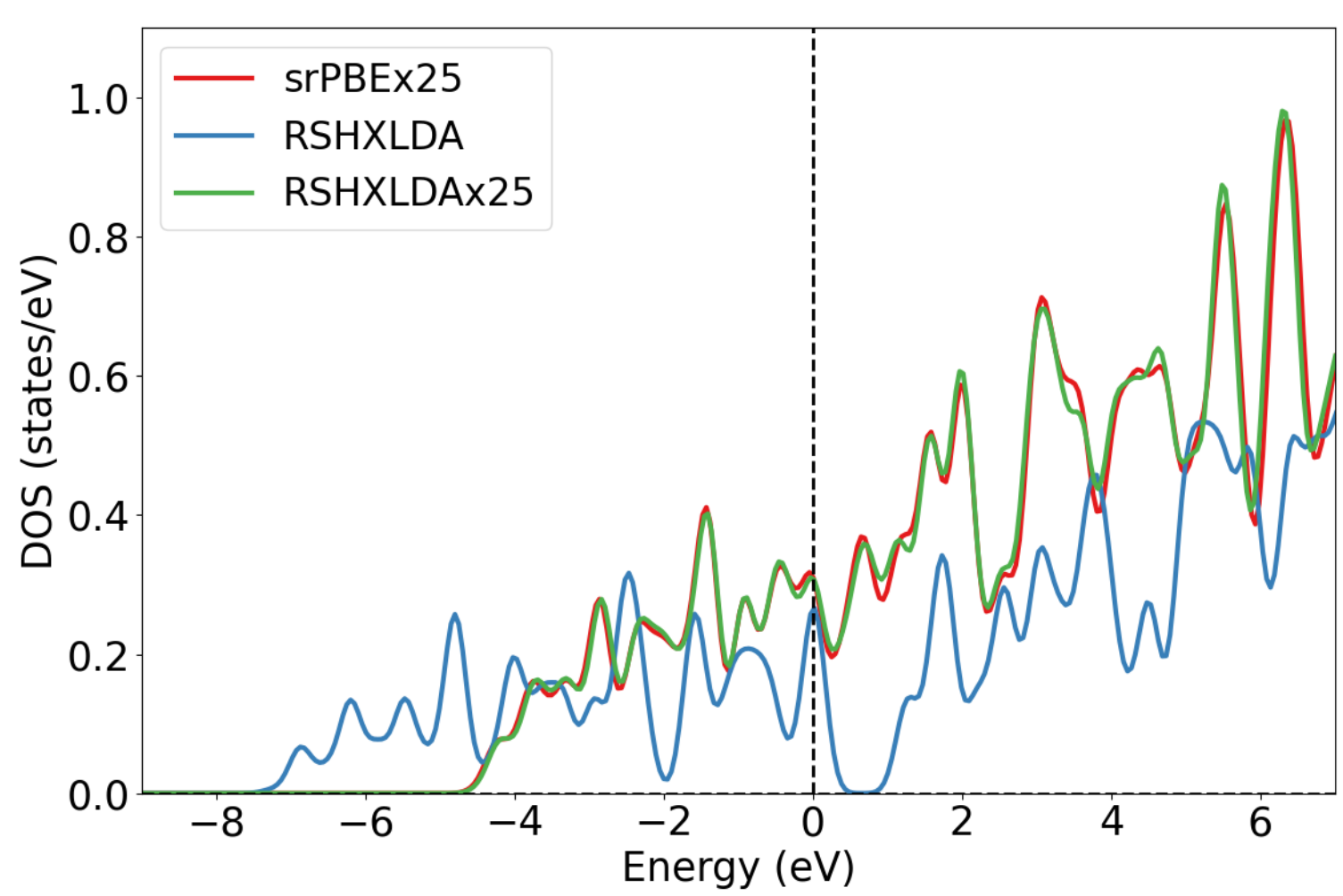

Figure S4: Density of states of bulk Na calculated using different functionals. LC-srPBEx25 retains states near the Fermi level, unlike RSHXLDA with 100% long-range EXX.

4 Energy Convergence

4.1 Metal bulk and surface

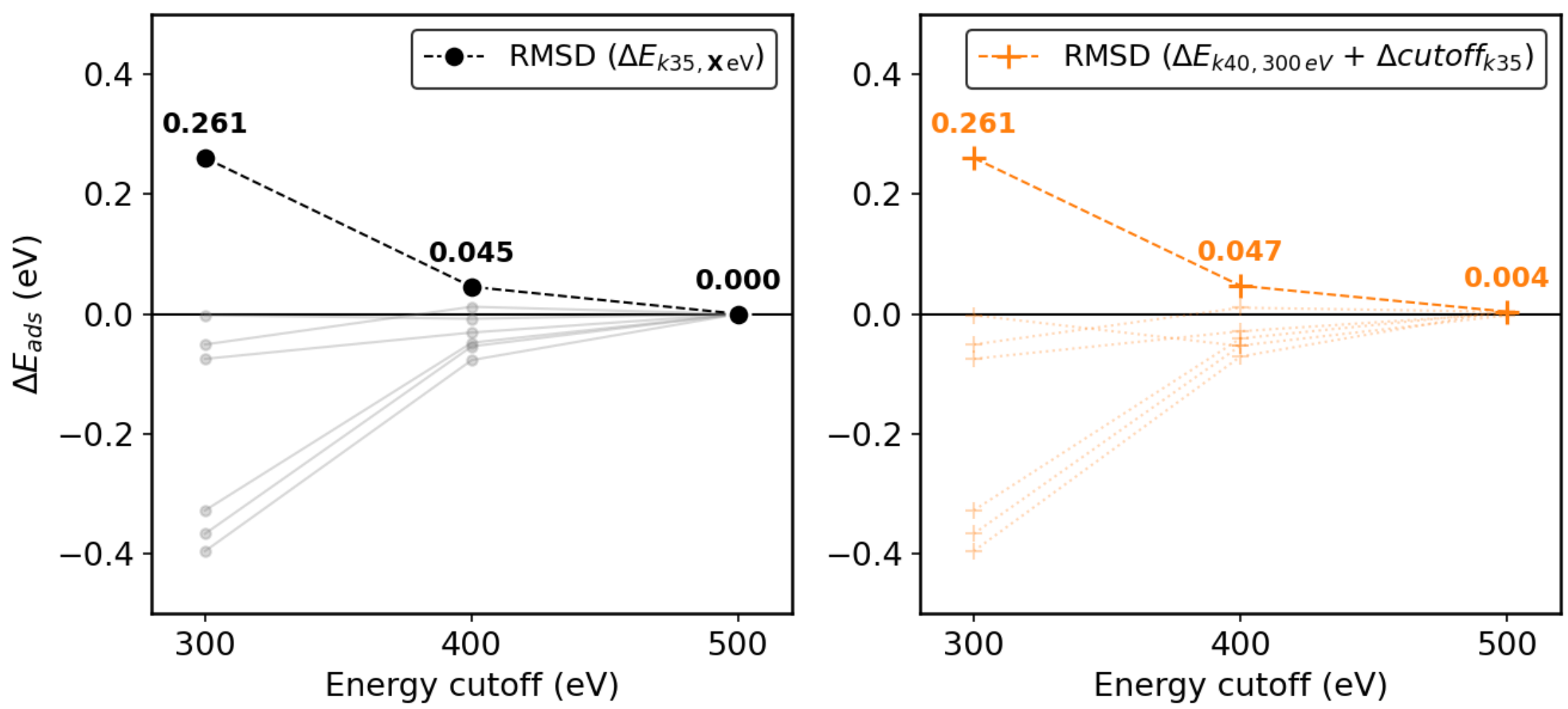

Figure S5: (Left) Convergence of adsorption energy on metal surfaces with respect to E_{cutoff} at k35 mesh, referenced to $\Delta E_{\mathrm{k35,\,500}}$. (Right) Validation of the correction term Δcutoff at different E_{cutoff}, referenced to $\Delta E_{\mathrm{k40,\,500}}$. The Δcutoff-corrected $\Delta E_{\mathrm{k40,\,300}}$ values closely match $\Delta E_{\mathrm{k40,\,500}}$, demonstrating the effectiveness of the correction. Semi-transparent symbols correspond to individual values, while opaque symbols indicate RMSDs relative to the references.

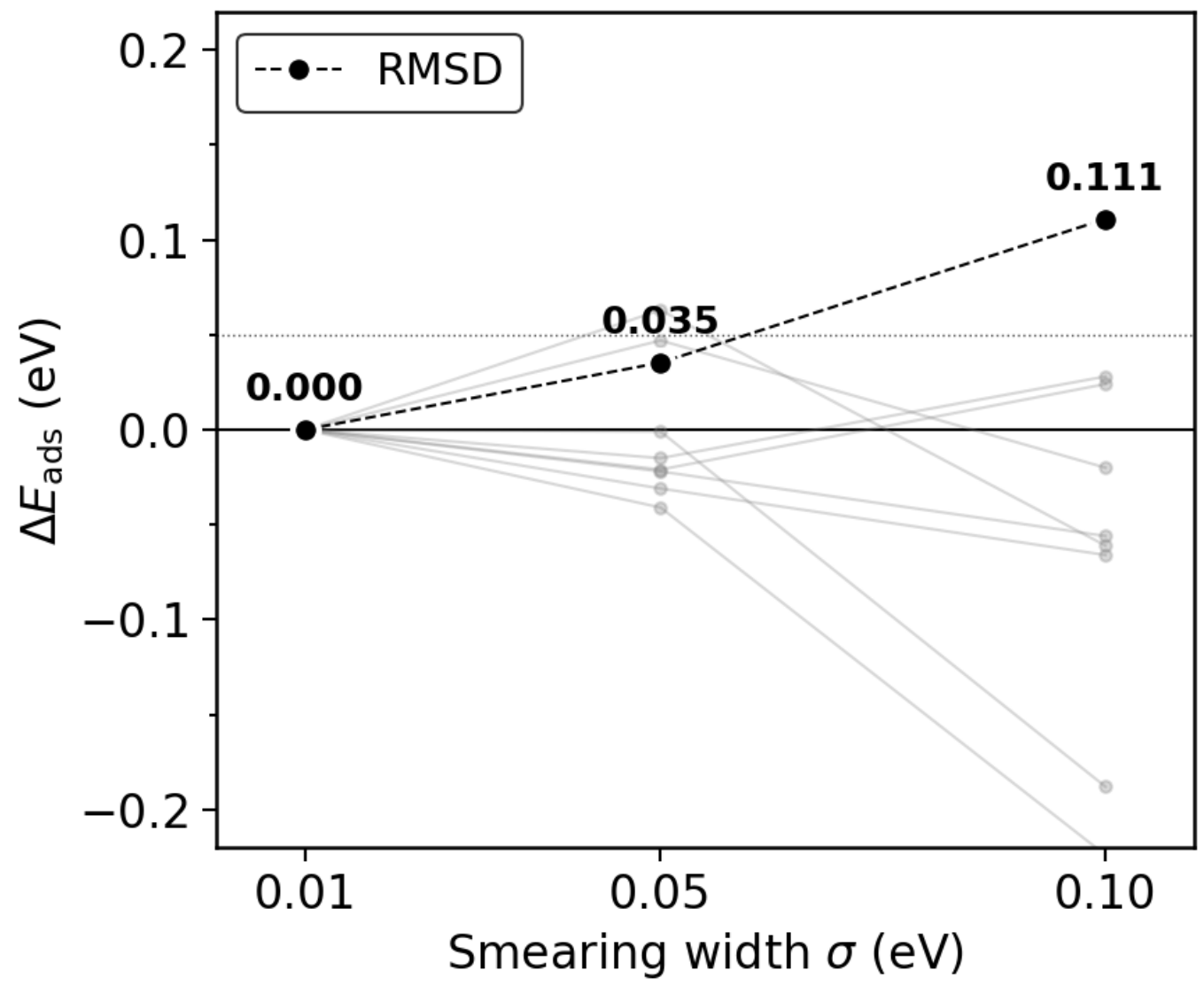

Figure S6: Convergence of adsorption energy on metal surfaces with respect to smearing width, referenced to values obtained with σ=0.01 eV. Semi-transparent symbols correspond to individual values, while opaque symbols indicate RMSDs relative to the reference.

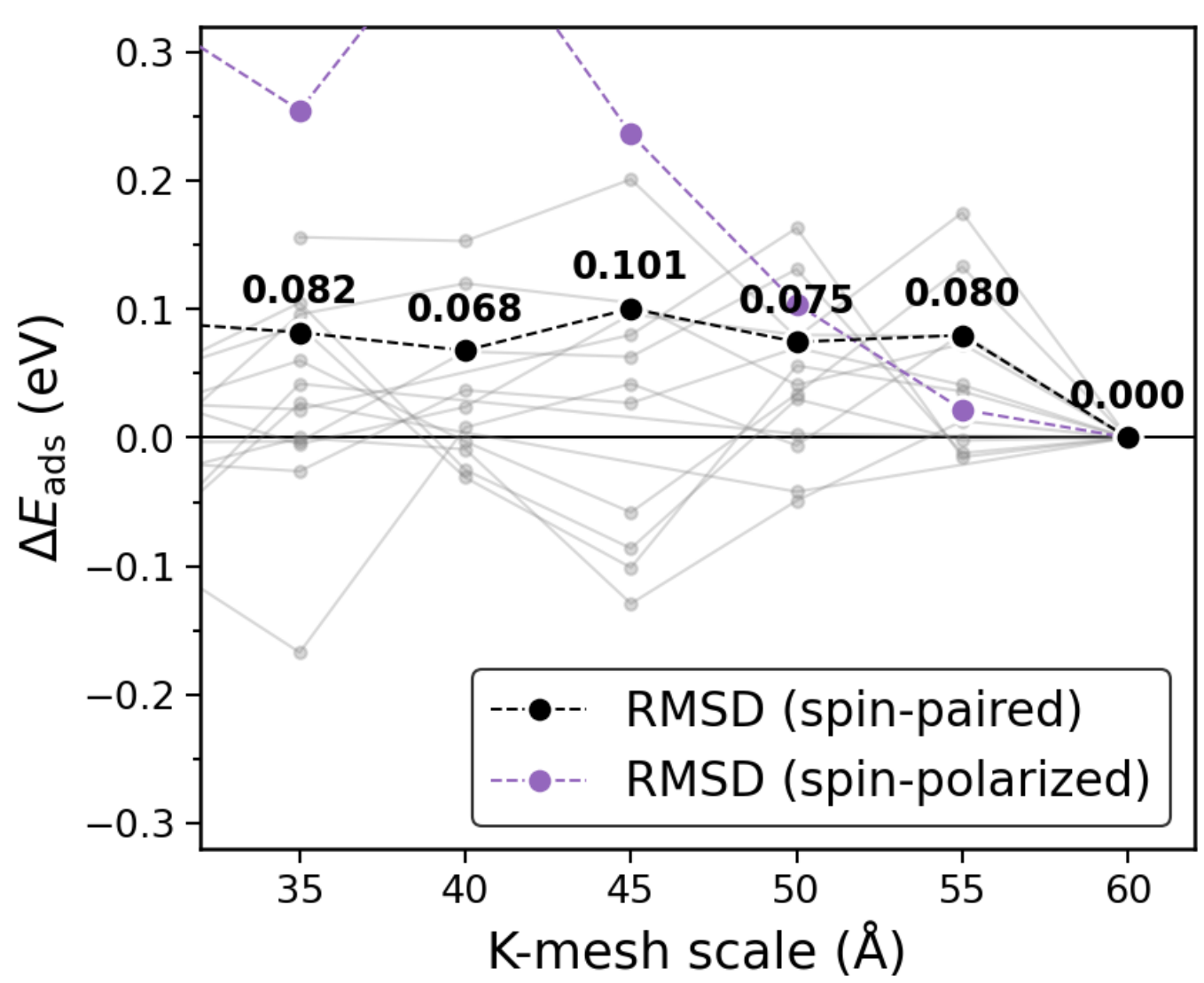

Figure S7: Convergence of adsorption energy on metal surfaces with respect to k-mesh scale, referenced to ΔE^{k60}. Semi-transparent symbols correspond to individual values, while opaque symbols indicate RMSDs relative to the references. Black symbols correspond to spin-paired results of non-magnetic systems, and purple symbols correspond to spin-polarized results of Ni(111) systems.

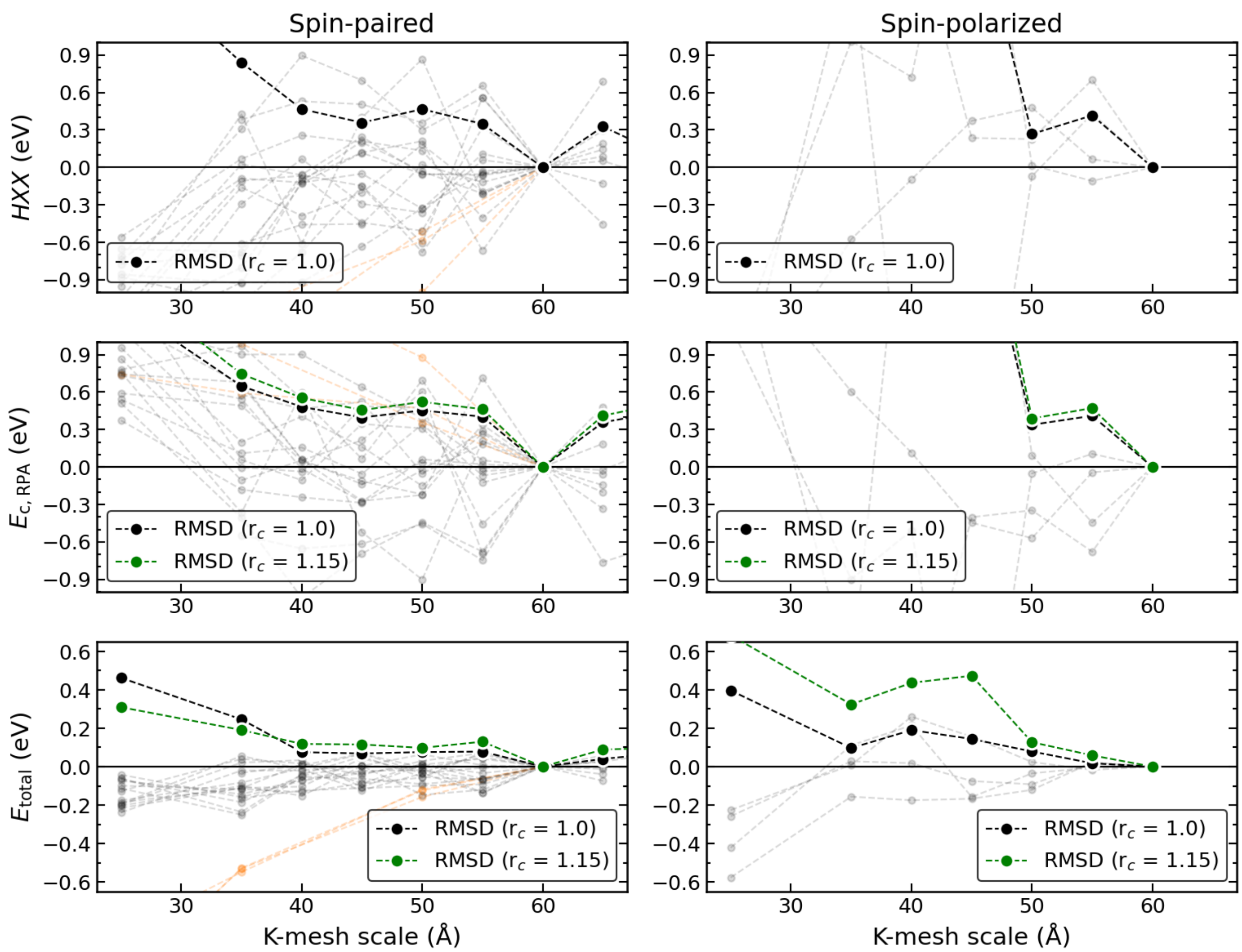

Figure S8: Convergence of HXX (top), RPA correlation energy (middle), and total energy (= HXX+$E_{\mathrm{c,RPA}}$) (bottom) of metal surface systems with respect to k-mesh scale, referenced to k60 values (r_c=1.0 or r_c=1.15). The left panels correspond to spin-paired results of non-magnetic systems, and the right pannels correspond to spin-polarized results of Ni(111) systems. Semi-transparent symbols correspond to individual values (r_c=1.0), while opaque symbols indicate RMSDs relative to the references. Orange symbols correspond to 3×3 slabs (r_c=1.0). Increasing the scaling factor from 1.0 to 1.15 worsens energy convergence.

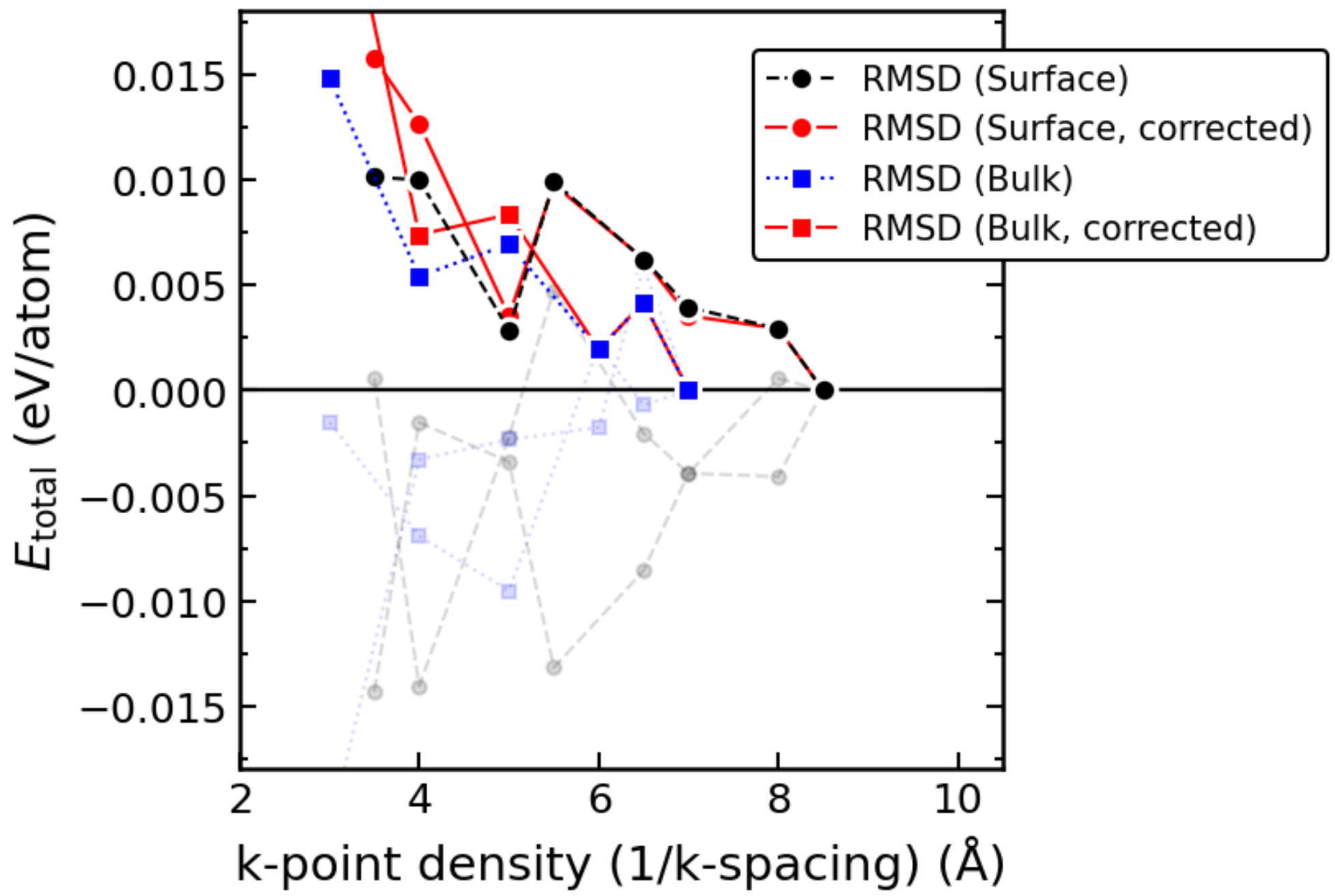

Figure S9: Convergence of total energy (= HXX+$E_{\mathrm{c,RPA}}$) of bulk and surface systems of Pd and Pt with respect to k-mesh scale, referenced to k-point densities of 7 Å (bulk) and 8.5 Å (= k65) (surface) (r_c=1.02). Semi-transparent symbols correspond to individual values, while opaque symbols indicate RMSDs relative to the references. Red symbols correspond to results with a correction to the non-RPA correlation part for partial occupancies (`ACFDT corr.` in VASP). The present work does not include this correction term.

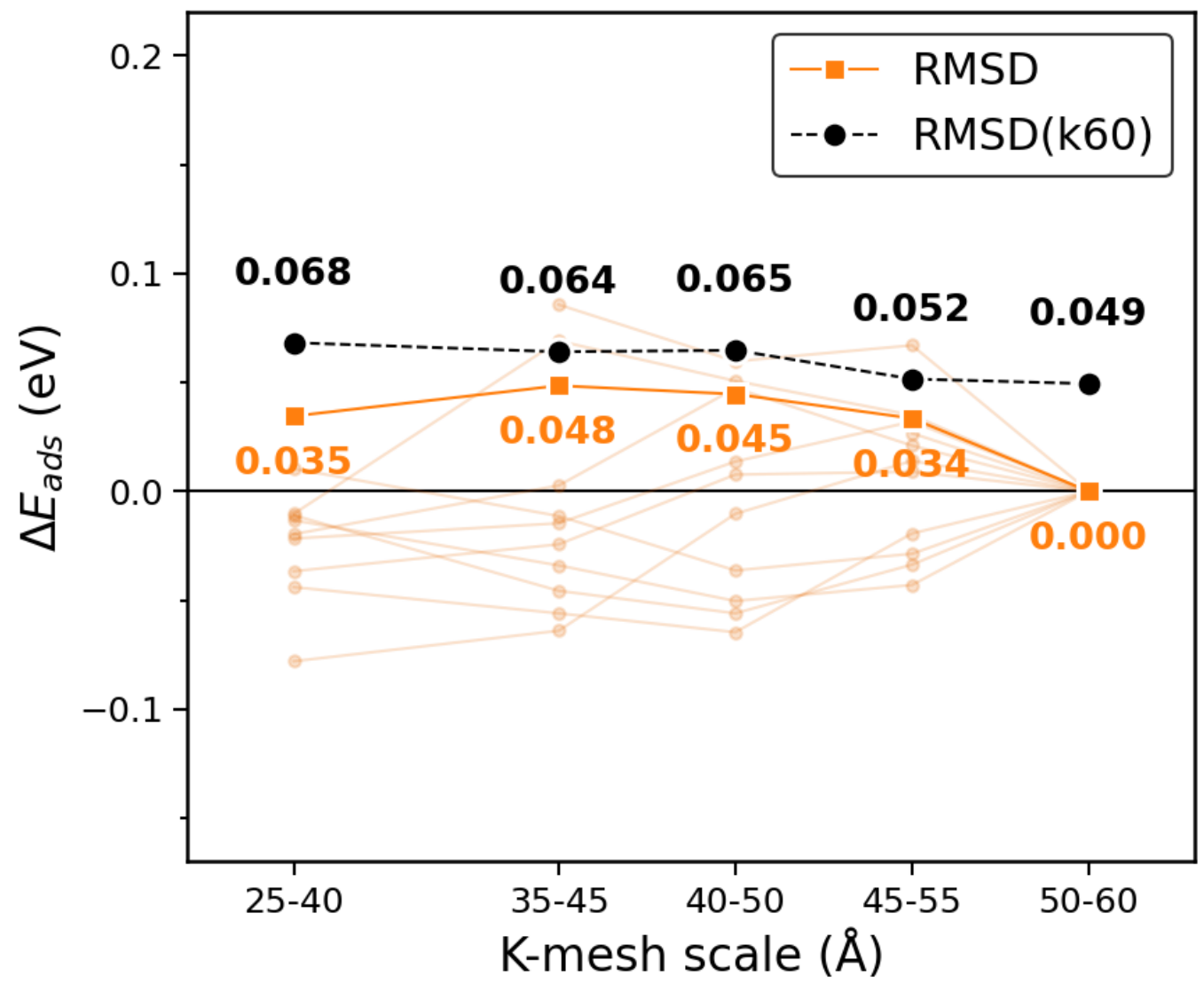

Figure S10: Convergence of averaged adsorption energy on metal surfaces with respect to k-mesh scales. Each y-axis value represents the averaged adsorption energy over three consecutive k-mesh scales (e.g., “25–40” values are averaged over k25, k35, and k40). The values are referenced to the averaged k50–k60 values (orange) or individual k60 values (black). Semi-transparent symbols correspond to individual values, while opaque symbols indicate RMSDs relative to the references.

4.2 Oxide surface

For the MgO surface, a 3×3 supercell with 2 layers was used for the baseline calculations. A 3×3 supercell with four layers (4L) was used to obtain the correction for surface thickness (Δlayer). A 5×5 supercell with two layers (2L) was used to obtain the correction for superell size (Δsupercell).

$$\Delta layer = \Delta E_{\text{k15, 400, 4L}} - \Delta E_{\text{k15, 400, 2L}}$$

$$\Delta supercell = \Delta E_{\text{k15, 400, 5}\times\text{5}} - \Delta E_{\text{k15, 400, 3}\times\text{3}}$$

$$\Delta E_{\text{high-k, high-E, 4L, 5}\times\text{5}} \approx (\Delta E_{\text{high-k, low-E}} + \Delta\text{cutoff}) + \Delta layer + \Delta supercell$$

The calculations for the Al_2O_3 surface used a 2×2 supercell with 12 layers, without corrections for slab thickness or supercell size. For the TiO_2 surface, a 1×3 supercell with 6 layers (6L) was used for the baseline calculations, and a 1×3 supercell with 7 layers (7L) was used

to obtain the correction for surface thickness (Δlayer).

$$\Delta layer = \Delta E_{\text{k1, 600, 7L}} - \Delta E_{\text{k1, 600, 6L}}$$

$$\Delta E_{\text{high-k, high-E, 7L}} \approx (\Delta E_{\text{high-k, low-E}} + \Delta\text{cutoff}) + \Delta layer$$

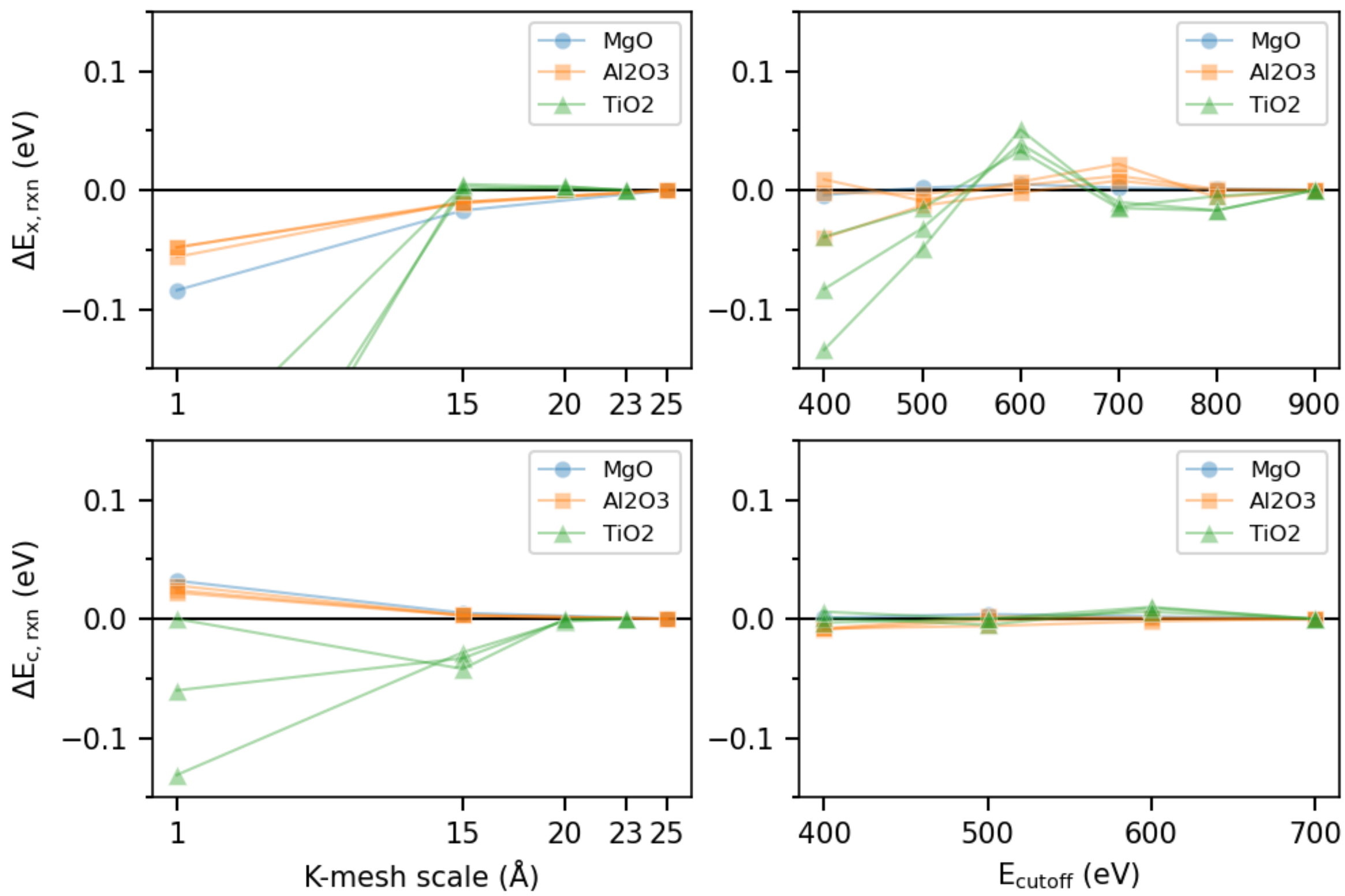

Figure S11: Convergence of surface reaction energy on the oxide surfaces. The top and bottom panels show the non-RPA correlation and RPA correlation contributions, respectively. Results are plotted with respect to k-mesh scale (left) and E_{cutoff} (right).

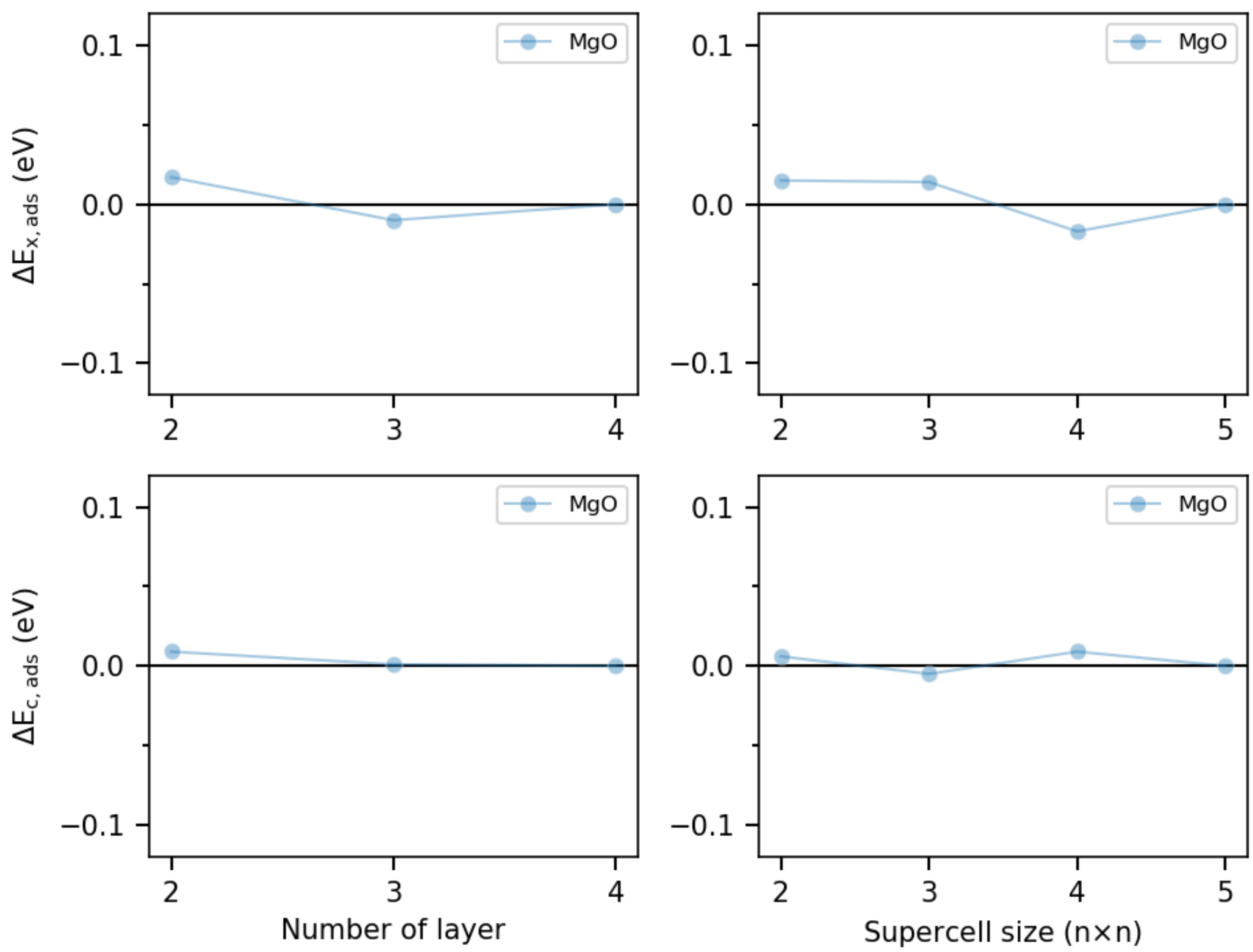

Figure S12: Convergence of adsorption energy on the MgO surface. The top and bottom panels show the non-RPA correlation and RPA correlation contributions, respectively. Results are plotted with respect to the number of surface layers (left) and the supercell size (right). A k-mesh scale of 15 Å was used. The surface structures have a supercell size of 3×3 (left) and consist of two layers (right).

4.3 Energy of infinitely dilute gas

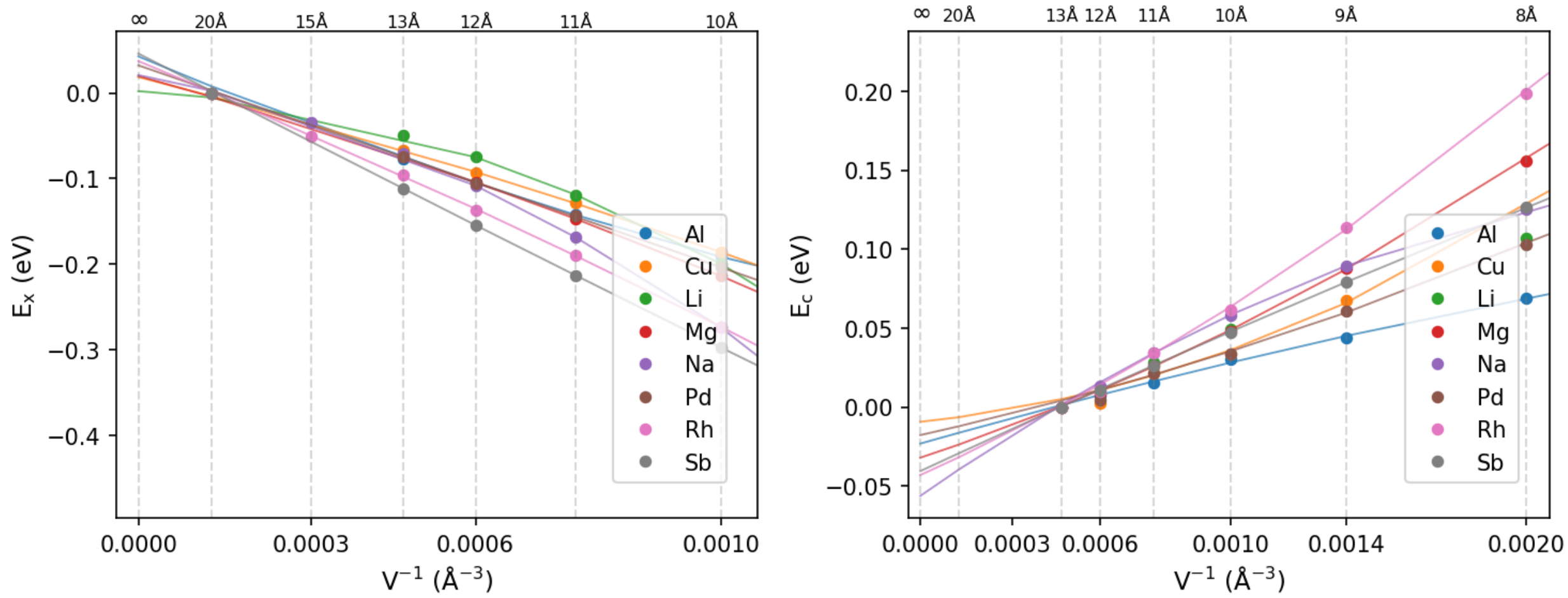

Figure S13: Volume dependence of E_{HXX} (a) and $E_{\mathrm{c,RPA}}$ (b) of gas species used for bulk properties, referenced to the energies at cell lengths of 20Å and 13Å, respectively. The solid lines represent the extrapolated energies using values at 10 – 13Å (E_x) or 8 – 11Å (E_c) cubic cells.

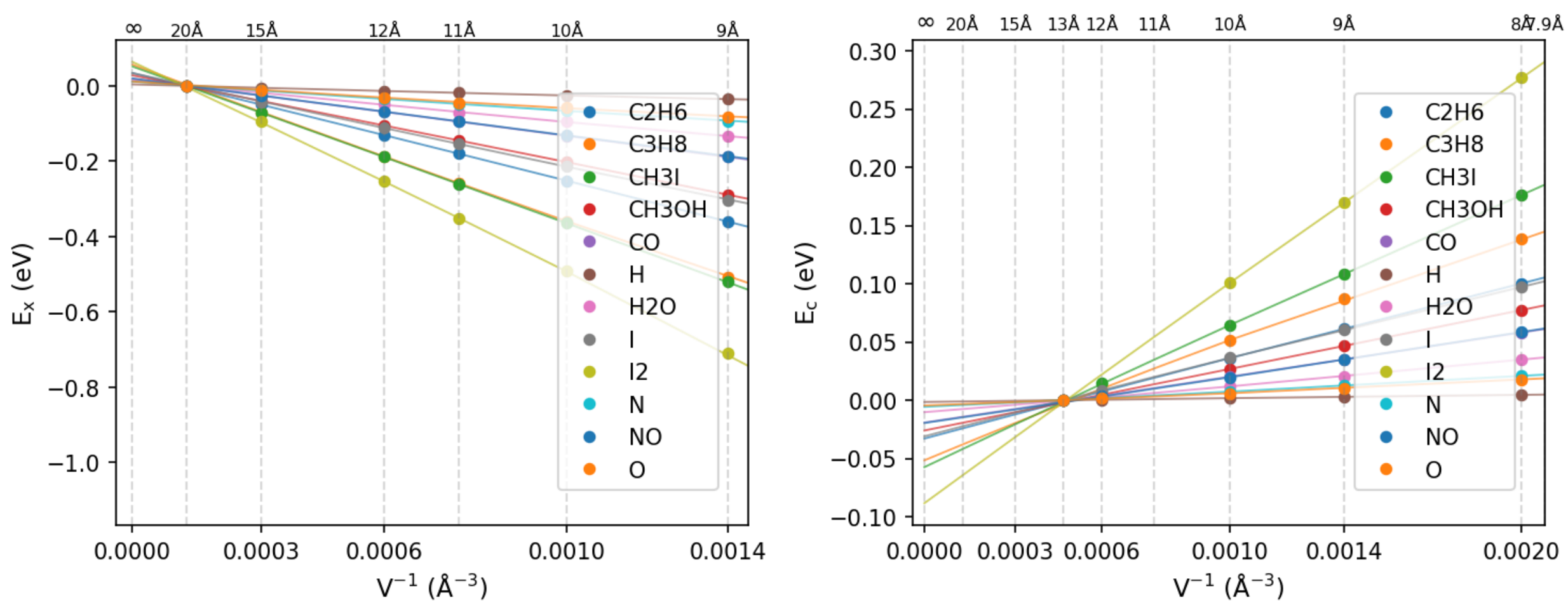

Figure S14: Volume dependence of E_{HXX} (a) and $E_{\mathrm{c,RPA}}$ (b) of adsorbates, referenced to the energies at cell lengths of 20Å and 13Å, respectively. The solid lines represent the extrapolated energies using values at 8 – 11Å (E_x) or 8 – 10Å (E_c) cubic cells.

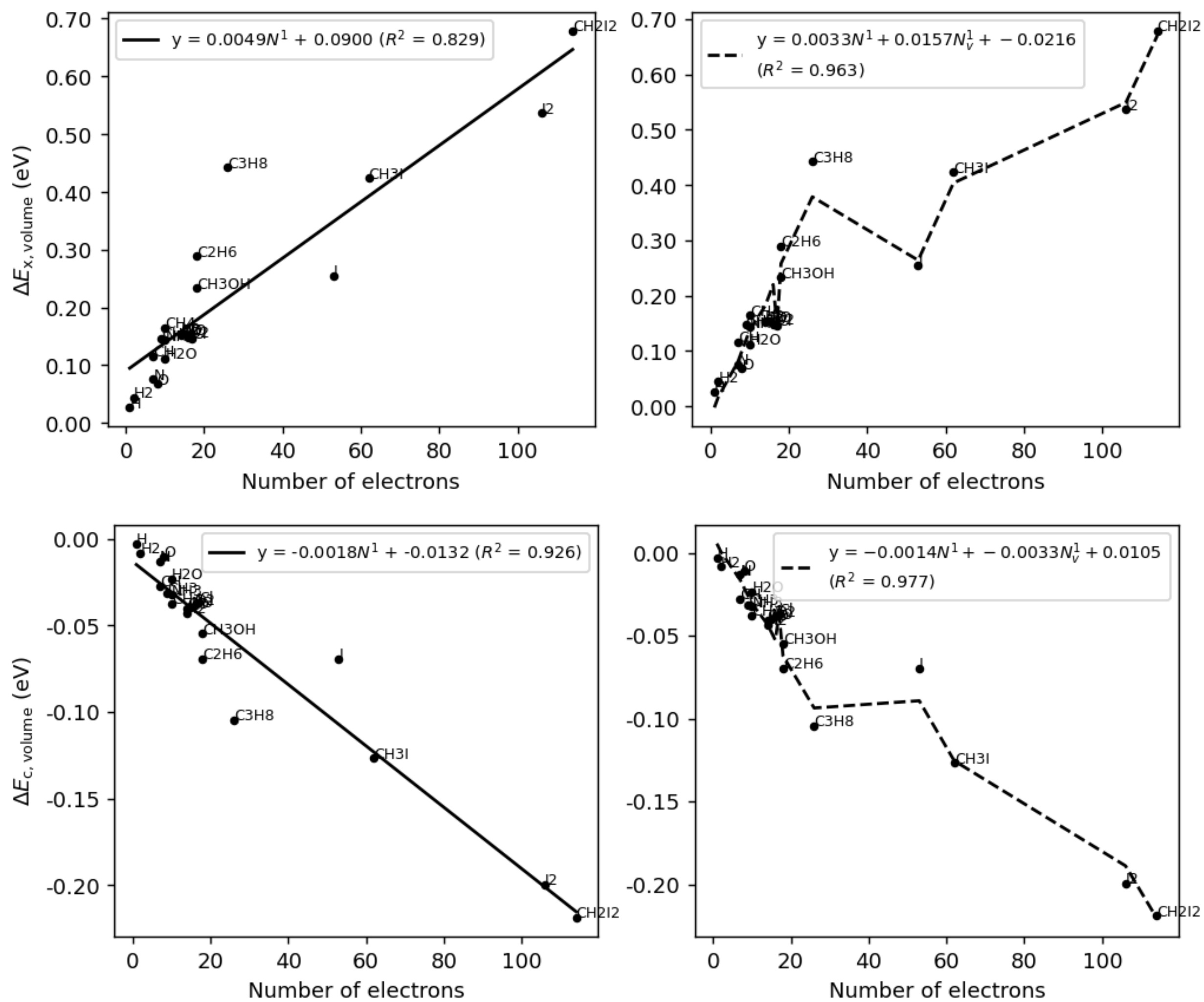

Figure S15: ΔE_{volume} ($= E_\infty - E_{10\text{Å}}$) at optRPA26 level as a function of the total number of electrons (N) in gas-phase species. Top panels show the exchange contribution $\Delta E_{\text{x,volume}}$, and bottom panels show the correlation contribution $\Delta E_{\text{c,volume}}$. Left panels include linear fits in N, while right panels include fits as functions of N and N_{v} (number of valence electrons).

5 Notes for RPA calculations

To reduce the cost of SCF cycles, the q-point grid can be reduced (`NKRED`-related tags) to pre-converge the wavefunction. In the next step, the calculation of unoccupied bands takes 500–2000 cpu hours, but only a single non-SCF step (one iteration) is required to determine the unoccupied bands. In the low-scaling RPA routine in VASP, the calculation of unoccupied

bands is done internally, but this can be avoided by manually setting the number of bands. Separating the calculation of unoccupied bands allows the use of parallelization settings that are not available during the RPA routine. The actual evaluation of RPA correlation energy only takes ∼500 cpu hours, but this step requires memory of 4000–6000 GB (which can be reduced by ∼1.5 by lowering `NTAUPAR` tag to adjust parallelization of imaginary time calculation). The large memory requirement can be handled by adjusting the number of CPUs allocated per task (via `--cpus-per-task` option in the Slurm workload manager), i.e., by using more nodes and less CPUs per node. Additional details can be found in the "Example_calculations" folder in the SI.

5.1 Miscellaneous

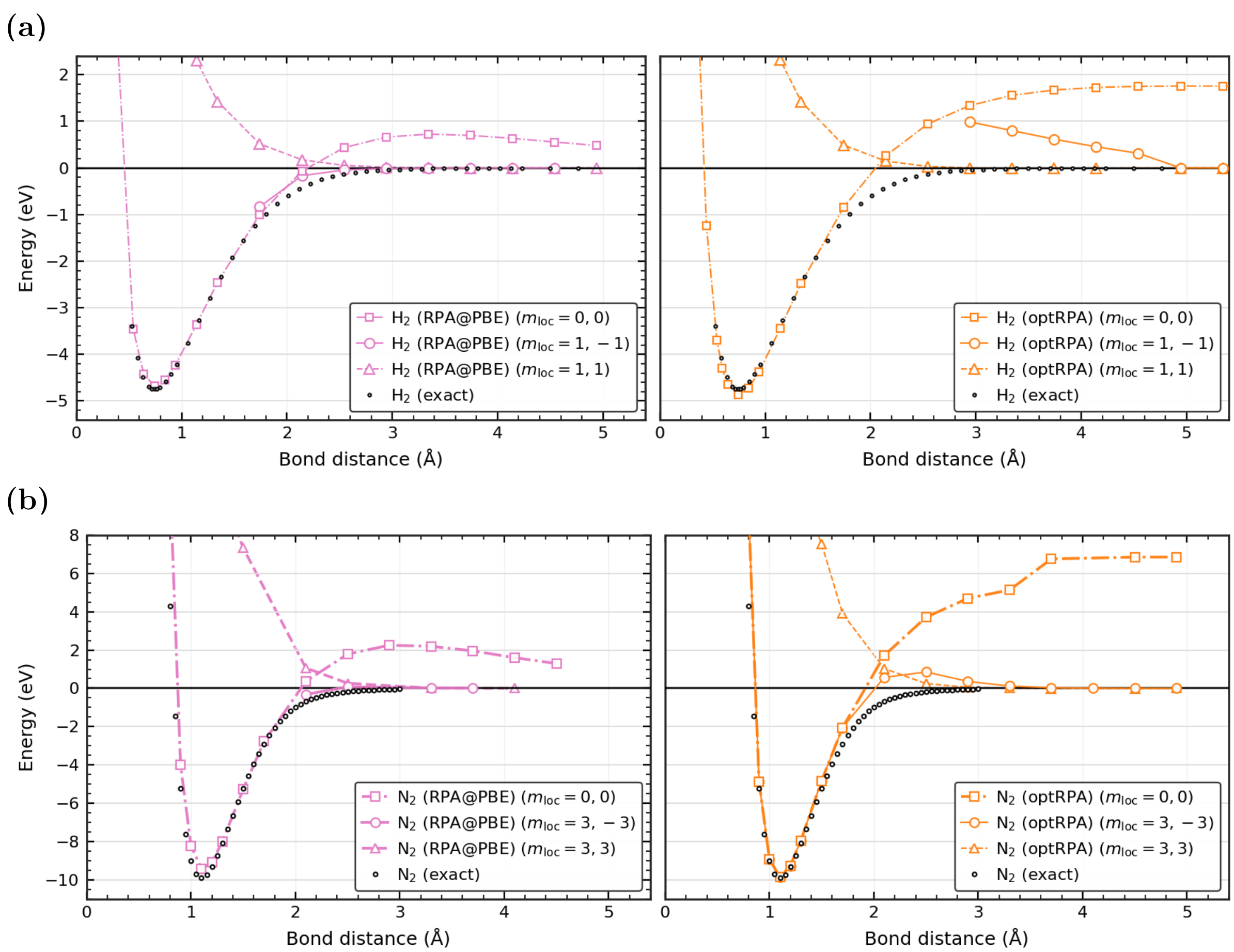

(c)

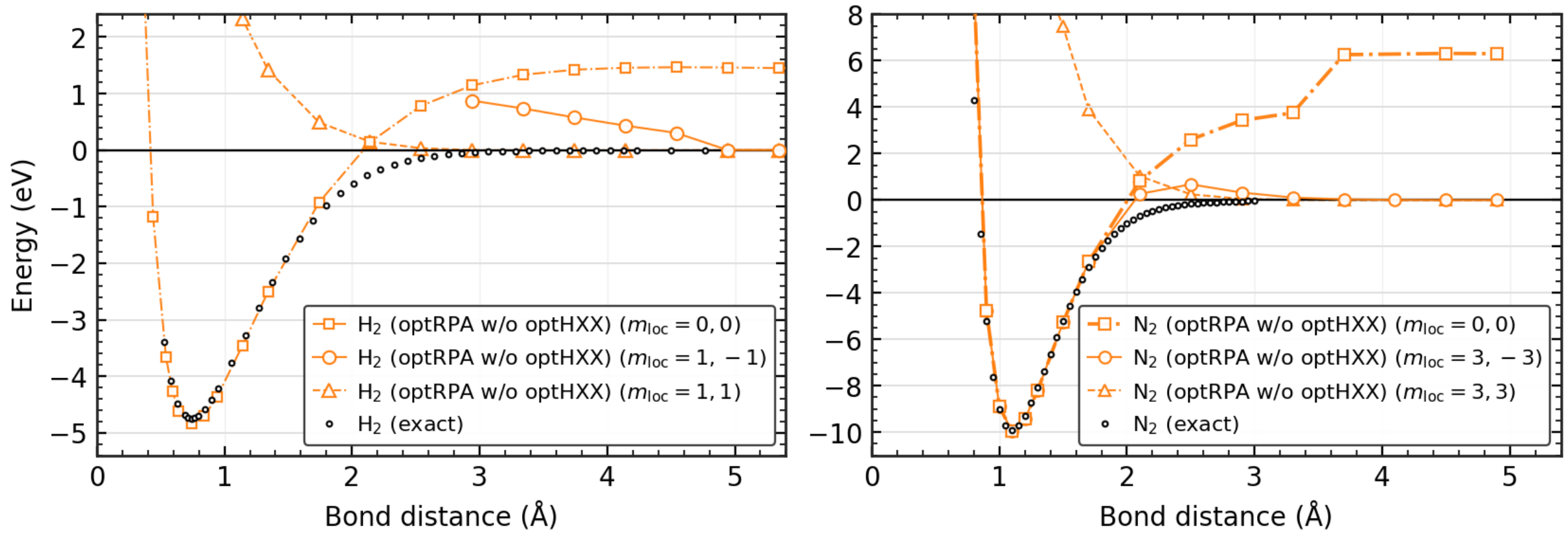

(d)

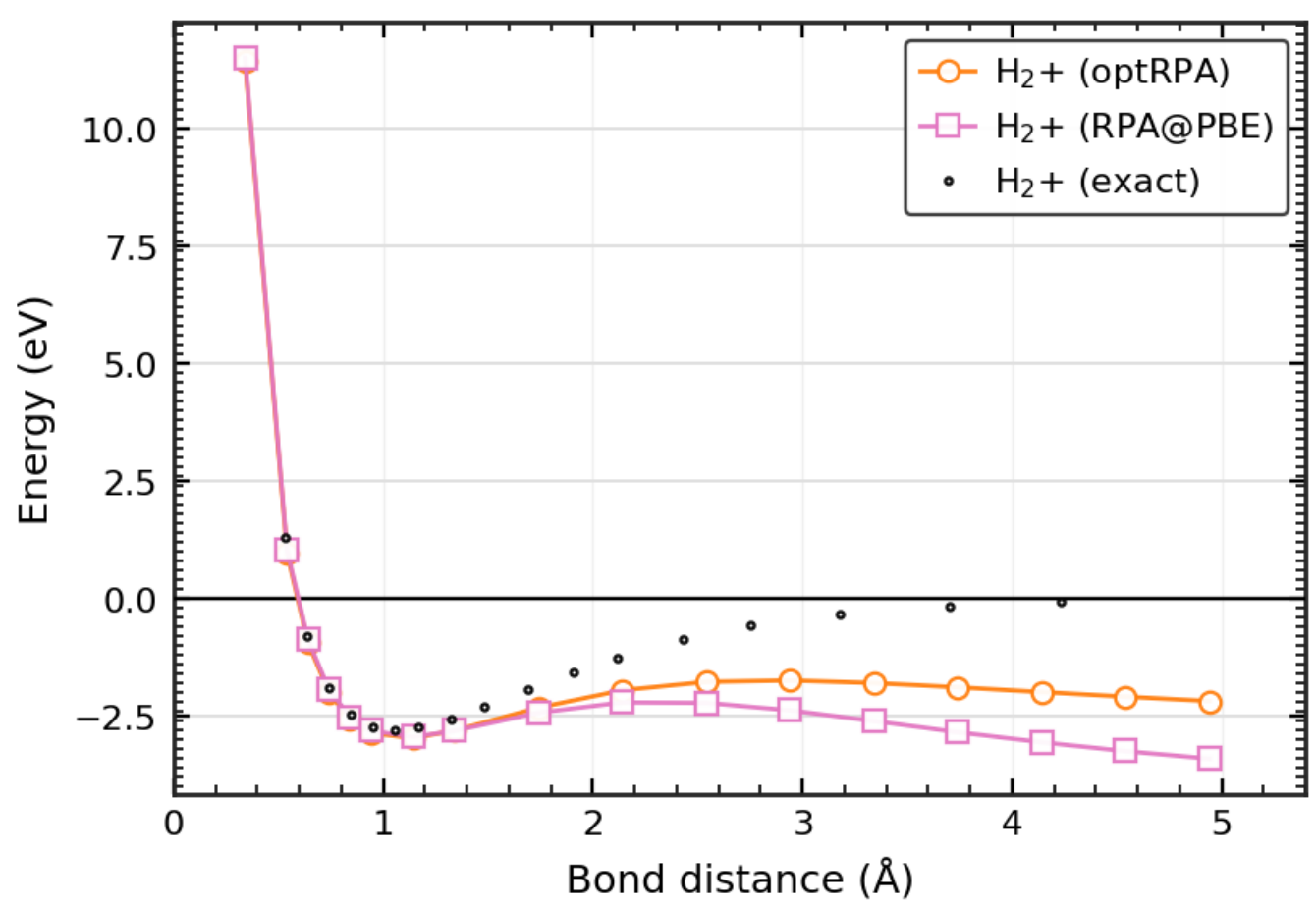

Figure S16: Dissociation curves for (a) H_2, (b) N_2, (c) H_2/N_2 using optRPA without optHXX ($r_c = 1.114$), and (d) ${H_2}^+$. Panels (a), (b), and (d) compare RPA@PBE and optRPA26 against the corresponding benchmark curves;[118–120] panel (c) shows the corresponding optRPA results without optHXX for H_2 and N_2. m_{loc} denotes the local spin moments of the two atoms; for H_2, $(0, 0)$, $(1, -1)$, and $(1, 1)$ correspond to singlet, broken-symmetry, and high-spin states, respectively. H_2 and N_2 dissociation probe static-correlation effects, whereas ${H_2}^+$ probes one-electron self-interaction and delocalization error.

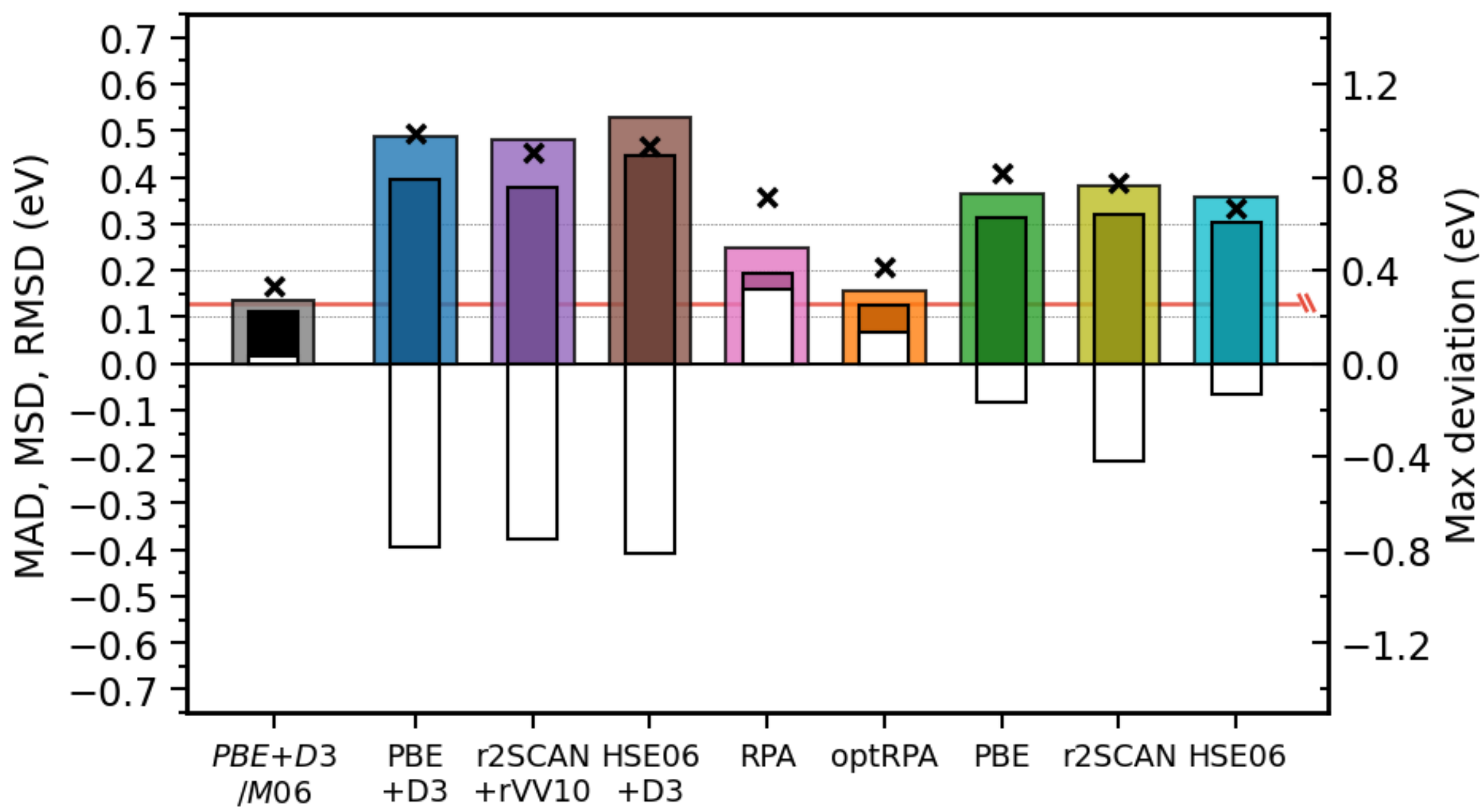

Figure S17: Deviations from ZPE-corrected experimental adsorption energies[41] on metals (Cu, Ru, Rh, Pd, Ir, Pt, and Ni) (n=27). *PBE+D3/M06*[41] refers to a hybrid scheme that combines cluster (M06 level) and periodic model (PBE+D3 level). Non-dissociative adsorption energies referenced to gas-phase adsorbates (e.g., O) as in Ref. 41. The adsorption energy values are defined on a per-adsorbate basis; for example, for O adsorption, the adsorption energy is ΔE in (a, c) or ΔE/2 in (b). Bars represent MAD (dark color), MSD (white color), and RMSD (light color), and × symbols indicate maximum deviations. Red horizontal lines denotes the transition metal chemical accuracy of 3 kcal/mol.

Table S4: RMSDs of least-squares energies obtained from slab-based adsorption energies ($E_{\text{slab+ads}} - E_{\text{slab}}$) and gas-phase adsorbate energies ($E_{\text{ads(g)}}$) to separate errors originating from slabs and gas-phase adsorbates. For $E_{\text{ads(g)}}$, each adsorbate is included multiple times based on its occurrence in the adsorption dataset (e.g., O adsorbate is used three times).

$\boldsymbol{E_i}$ (in Eq. (11))	***PBE+D3/M06***	**PBE +D3**	**r2SCAN +rVV10**	**HSE06 +D3**	**RPA**	**optRPA26**
$E_{\text{slab+ads}} - E_{\text{slab}}$	0.119	0.175	0.175	0.277	0.172	0.108
$E_{\text{ads(g)}}$	0.051	0.172	0.139	0.135	0.115	0.050

$\boldsymbol{E_i}$ (in Eq. (11))	**PBE**	**r2SCAN**	**HSE06**
$E_{\text{slab+ads}} - E_{\text{slab}}$	0.175	0.170	0.267
$E_{\text{ads(g)}}$	0.164	0.126	0.127
